# Balões Revestidos com Paclitaxel versus Stents Farmacológicos na Doença Arterial Coronariana de Pequenos Vasos: Revisão Sistemática e Metanálise

**DOI:** 10.36660/abc.20250069

**Published:** 2025-11-26

**Authors:** Marília Oberto da Silva Gobbo, Renan Yuji Ura Sudo, Ancy Jenil Franco, Mable Pereira, Tanize Louize Milbradt, Zeynep Eylul Bakir, Iago Zang Pires, Stephen Akinfenwa, Gustavo Luís Agostini, Denise Pellegrini, Paulo R. A. Caramori

**Affiliations:** 1 Pontifícia Universidade Católica do Rio Grande do Sul Porto Alegre RS Brasil Pontifícia Universidade Católica do Rio Grande do Sul, Porto Alegre, RS – Brasil; 2 Universidade Federal da Grande Dourados Dourados MS Brasil Universidade Federal da Grande Dourados, Dourados, MS – Brasil; 3 McLaren Bay Region Internal Medicine Department Bay City Michigan EUA McLaren Bay Region, Internal Medicine Department, Bay City, Michigan – EUA; 4 Lincoln American University School of Medicine Georgetown Guiana Lincoln American University School of Medicine, Georgetown – Guiana; 5 Universidade Federal de Santa Maria Santa Maria RS Brasil Universidade Federal de Santa Maria, Santa Maria, RS – Brasil; 6 University of Rome La Sapienza Rome Lazio Itália University of Rome La Sapienza, Rome, Lazio – Itália; 7 University of Connecticut Storrs EUA University of Connecticut, Storrs – EUA; 8 Tacchini Hospital Bento Gonçalves RS Brasil Tacchini Hospital, Bento Gonçalves, RS – Brasil; 9 Hospital São Lucas PUCRS Porto Alegre RS Brasil Hospital São Lucas da PUCRS, Porto Alegre, RS – Brasil

**Keywords:** Stents Farmacológicos, Doença da Artéria Coronariana, Intervenção Coronária Percutânea

## Abstract

**Fundamento:**

Os stents farmacológicos (SF) permanecem como padrão terapêutico na intervenção coronariana percutânea em casos de doença arterial coronariana de pequenos vasos (DACPV). Balões revestidos com paclitaxel (BRP) têm surgido como alternativa promissora, com estudos recentes indicando desfechos favoráveis em curto e médio prazos, embora a maioria apresente limitações quanto ao tamanho amostral.

**Objetivo:**

Comparar os desfechos clínicos e angiográficos entre SF e BRP em pacientes com DACPV.

**Métodos:**

Foram realizadas buscas nas bases de dados PubMed, Embase, Cochrane Library e ClinicalTrials.gov por estudos que comparassem BRP e SF em DACPV, definida como diâmetro de referência do vaso < 3,0 mm. Realizou-se metanálise com modelo de efeitos aleatórios, apresentando os resultados como diferença média (DM) ou razão de risco (RR). A significância estatística foi considerada para p < 0,05. A heterogeneidade foi avaliada por meio dos testes qui-quadrado, Tau e Tau^2^. O protocolo da revisão foi registrado na PROSPERO (CRD42024506502).

**Resultados:**

Foram incluídos 12 estudos, totalizando 17.441 pacientes (média de idade entre 58 e 73 anos), com follow-up clínico variando entre 8 e 36 meses. Todos os estudos avaliaram BRP. Não foram observadas diferenças significativas entre BRP e SF em relação à revascularização da lesão-alvo (RR, 1,24; IC 95%, 0,82-1,85; p = 0,30), perda tardia de lúmen (DM, –0,09 mm; IC 95%, –0,41 a 0,23; p = 0,57), eventos cardiovasculares adversos maiores (RR, 1,01; IC 95%, 0,76-1,33; p = 0,95), mortalidade por todas as causas (RR, 0,81; IC 95%, 0,50-1,31; p = 0,39), mortalidade cardiovascular (RR, 1,74; IC 95%, 0,78-3,89; p = 0,17) e infarto do miocárdio (RR, 0,76; IC 95%, 0,46-1,27; p = 0,30).

**Conclusões:**

A angioplastia com BRP apresentou desfechos clínicos e angiográficos comparáveis aos observados com SF na DACPV, reforçando sua viabilidade como alternativa segura e eficaz em pacientes selecionados.

## Graphical abstract

**Central Illustration:**
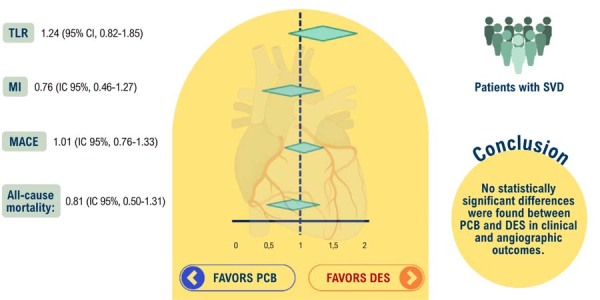
Paclitaxel-Coated Balloons versus Drug-Eluting Stents in Small Vessel Coronary Artery Disease: A Systematic Review and Meta-Analysis

DES: drug-eluting stent; MACE: major adverse cardiovascular events; MI: myocardial infarction; PCB: paclitaxel-coated balloon; SVD: small vessel disease; TLR: target lesion revascularization.

## Introdução

A doença arterial coronariana de pequenos vasos (DACPV) é comum em pacientes submetidos à intervenção coronariana percutânea (ICP), representando até 50% de todos os procedimentos realizados anualmente.^1^ O tratamento da DACPV com *stents* farmacológicos (SF) apresenta desafios específicos, incluindo maior risco de dissecção, ruptura do vaso e reestenose, além da necessidade de terapia antiplaquetária dupla (TAD) prolongada após a implantação do *stent* .^2^

Embora os SF tenham demonstrado superioridade em relação à angioplastia com balão convencional, os balões farmacológicos (BF) surgiram como alternativa promissora em contextos anatômicos específicos, como na DACPV.^3^ Os BF consistem em cateteres-balão revestidos com agentes antiproliferativos — geralmente paclitaxel, sirolimus ou análogos limus — liberados na parede do vaso durante a insuflação do balão.^4^ A insuflação costuma durar 30-60 segundos. Essa estratégia evita a implantação de material estranho permanente, o que pode reduzir reações inflamatórias tardias e diminuir o risco de reestenose e trombose. A ausência de arcabouço metálico e polímero durável contribui para a preservação da anatomia vascular e da função vasomotora, minimizando distúrbios hemodinâmicos e, potencialmente, reduzindo a necessidade de TAD prolongada.^5^

O paclitaxel é citotóxico, mas atinge concentrações citotóxicas na parede do vaso apenas nas primeiras horas após a aplicação do BF. Posteriormente, sua ação se assemelha à do sirolimus. Devido à sua alta lipofilicidade, o paclitaxel apresenta maior retenção na parede vascular, proporcionando inibição mais sustentada da proliferação neointimal em comparação aos fármacos à base de limus. Isso pode conferir uma vantagem a longo prazo aos balões revestidos com paclitaxel (BRP) em relação aos dispositivos revestidos com sirolimus.^6 , 7^

Apesar do consenso entre especialistas quanto à viabilidade da angioplastia com BF como alternativa aos SF na DACPV, as evidências oriundas de ensaios clínicos randomizados (ECRs) ainda são limitadas, principalmente em razão do pequeno tamanho amostral.^8 - 11^ Diante disso, realizamos uma revisão sistemática e metanálise para sintetizar os dados disponíveis que comparam os desfechos clínicos dessas duas estratégias terapêuticas.

## Métodos

### Critérios de elegibilidade

Foram considerados elegíveis os estudos que atendessem aos seguintes critérios: ECRs ou estudos de coorte não randomizados; estratégias de ICP envolvendo BFs ou SFs; e inclusão de pacientes com DACPV (diâmetro de referência do vaso < 3,0 mm). Os estudos foram incluídos apenas se relatassem pelo menos um desfecho clínico relevante.

Foram excluídos estudos com comparadores inadequados, diâmetro de vaso de referência ≥ 3 mm, *follow-up* inferior a 6 meses, texto completo indisponível ou publicações em idiomas diferentes do inglês.

### Estratégia de busca e extração de dados

Esta revisão sistemática e metanálise seguiu as recomendações da Cochrane Collaboration e foi relatada conforme os itens do Preferred Reporting Items for Systematic Reviews and Meta-Analyses (PRISMA) ( Tabela S1 ).^12 , 13^ O protocolo da revisão foi prospectivamente registrado na base PROSPERO em 25 de janeiro de 2024 sob o código CRD42024506502.

As buscas foram conduzidas por dois revisores nas bases PubMed, Embase e Cochrane Library, desde suas criações até janeiro de 2024, utilizando os termos drug-coated balloon, drug-eluting stent e coronary small vessel. A estratégia completa de busca está disponível no material suplementar. As listas de referências também foram examinadas em busca de estudos relevantes. O banco ClinicalTrials.gov foi consultado para identificar ensaios adicionais não recuperados na busca principal.

As características dos estudos, dados basais e desfechos clínicos foram extraídos de forma independente por 4 revisores, seguindo critérios predefinidos e avaliações de qualidade. As divergências foram resolvidas por meio de discussão com outros 2 revisores, e, quando necessário, com a participação dos revisores seniores.

### Desfechos e subanálises

O desfecho primário foi a incidência de RLA motivada por critérios clínicos, no maior tempo de *follow-up* disponível. O principal desfecho angiográfico secundário foi a PTL. Outros desfechos de interesse incluíram eventos cardiovasculares adversos maiores (ECAM), mortalidade por todas as causas, mortalidade cardiovascular (CV) e infarto agudo do miocárdio (IAM). Os dados detalhados dos desfechos de cada estudo estão apresentados no Material Suplementar (Tabela S2) .

### Avaliação da qualidade

Os estudos de coorte não randomizados foram avaliados utilizando a Newcastle-Ottawa Scale (NOS).^14^ Essa ferramenta atribui até 9 estrelas com base em 3 domínios: seleção dos participantes, comparabilidade dos grupos e determinação dos desfechos ( Tabela S3 ). Os ECRs foram avaliados por meio da ferramenta Cochrane Risk of Bias 2.0 (RoB 2), que classifica os estudos como de “baixo risco de viés”, “algumas preocupações” ou “alto risco de viés” em 5 domínios: processo de randomização, desvios das intervenções planejadas, dados ausentes dos desfechos, mensuração dos desfechos e seleção dos resultados relatados ( Figura S4 ).^15^

A certeza da evidência foi avaliada por 2 revisores independentes (R.S. e M.G.) utilizando o sistema GRADE ( *Grading of Recommendations, Assessment, Development and Evaluation* ), com níveis que variam de alta a muito baixa ( Tabela S5 ).^16^ A presença de viés de publicação foi explorada por meio de análise gráfica em funil com estimativas pontuais ponderadas pelo tamanho dos estudos e teste de Egger.

### Análise estatística

Os dados foram sintetizados por meio de metanálise com modelo de efeitos aleatórios, utilizando o estimador de máxima verossimilhança restrita. Esse modelo foi escolhido para considerar possíveis heterogeneidades clínicas, metodológicas e estatísticas, reconhecendo que o verdadeiro efeito da intervenção pode variar entre os estudos.^17 , 18^ Os desfechos dicotômicos foram expressos como razões de risco (RR) e os desfechos contínuos como diferenças médias (DM). Análises de subgrupos compararam os desfechos entre ECRs e estudos observacionais, levando em conta possíveis interações qualitativas e quantitativas. A significância estatística foi definida por um IC 95% e valor de p < 0,05. A heterogeneidade foi avaliada pelos testes qui-quadrado, Tau e Tau^2^. Para evitar interpretações equivocadas decorrentes de um limiar fixo para o I^2^, a heterogeneidade também foi analisada em relação ao intervalo de predição (PI).^18 , 19^ Todas as análises estatísticas foram realizadas no software R, versão 4.4.1 (R Foundation for Statistical Computing).^20^

## Resultados

### Estratégia de busca e características basais dos estudos incluídos

**Figura 1:**
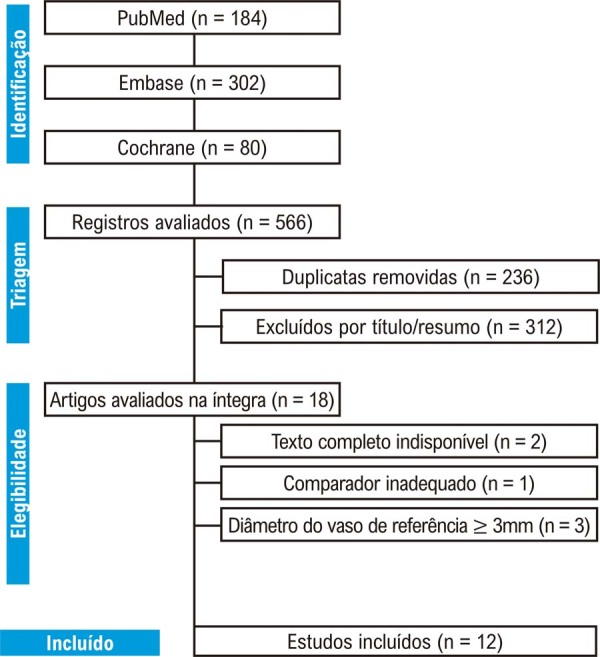
– Fluxograma PRISMA para identificação, triagem e inclusão dos estudos. PRISMA: Preferred Reporting Items for Systematic Reviews and Meta-Analyses.

A busca inicial identificou 566 registros. Após a remoção de 236 duplicatas, 312 artigos foram excluídos com base no título e resumo ( Figura 1 ). Um total de 18 artigos foi submetido à avaliação do texto completo. Destes, 2 foram excluídos por indisponibilidade do texto completo, 1 por utilizar comparador inadequado e 3 por incluir pacientes com diâmetro de vaso de referência ≥ 3 mm. Foram incluídos 12 estudos, totalizando 17.441 pacientes com DACPV.^21 - 32^ As publicações ocorreram entre 2010 e 2024, com *follow-up* médio variando entre 8 e 36 meses. Todos os BFs utilizados eram revestidos com paclitaxel. Os nomes comerciais dos BRPs e dos SFs utilizados nos estudos individuais estão listados no Material Suplementar (Tabela S6) .

As características dos estudos e intervenções incluídos estão resumidas nas Tabelas 1 e 2 . Na linha de base, a população era predominantemente masculina, com média de idade de 64 anos e alta prevalência de diabetes e hipertensão. O diâmetro médio do vaso de referência foi de 2,19 mm. A artéria descendente anterior esquerda foi o local mais comum das lesões-alvo. Quase metade dos pacientes apresentava doença multivascular. A fração de ejeção do ventrículo esquerdo (FEVE) não foi relatada na metade dos estudos; nos estudos que forneceram esses dados, os valores da FEVE estavam dentro da faixa compatível com FE preservada.

**Tabela 1:** – Características clínicas basais dos estudos incluídos

Estudo/Característica	Cortese, et al., 2010^21^	Latib, et al., 2012^22^	Sinaga, et al., 2016^27^	Giannini, et al., 2017^23^	Jeger, et al., 2018^28^	Sim, et al., 2018^24^	Tang, et al., 2018^29^	Cortese, et aI., 2020^31^	Silverio, et al., 2020^30^	Tsai, et al., 2022^26^	Kawai, et al., 2022^25^	Liu, et al., 2024^32^
Tamanho da amostra, n	28/29	90/92	172/163	90/91	382/376	87/200	116/110	118/114	1154/13634	47/59	19/23	129/118
Tipo de estudo	RCT	RCT	Coorte retrospectiva	Coorte retrospectiva	ECR	Coorte retrospectiva	ECR	ECR	Coorte prospectiva	Coorte retrospectiva	ECR	ECR
*Follow-up* clínico, meses	9	24	12	12	12	12	12	12	36	12	8	12
Idade, anos	68/67	65/66	61/61	65/66	67/68	58/61	60/60	64/66	68/69	65	69/73	60/60
Sexo masculino, %	79/76	80/77	77/72	80/78	77/70	81/77	77/77	77/70	70/66	83/81	79/74	73/70
Diabetes, %	46/37	43/38	51/49	43/40	32/34	63/54	39/43	38/35	35/38	49/54	26/34	35/38
Hiperlipidemia, %	60/45	78/79	70/72	79/78	68/69	80/76	52/50	60/55	59/50	85/71	79/82	45/54
Hipertensão, %	75/69	80/81	72/69	78/81	84/88	79/68	67/78	65/66	68/64	93/83	89/91	73/75
Tabagismo atual, %	NA	16/10	30/44	43/33	21/19	48/29	29/32	19/16	12/16	44/35	52/52	21/21
IAM prévio, %	17/20	51/35	28/27	51/58	41/35	56/57	22/25	28/39	31/24	10/11	10/21	25/22
DAC estável, %	46/44	75/78	22/34	75/81	70/73	NA	31/30	53/56	32/23	87/81	100/100	20/25
Angina instável, %	53/55	24/21	49/38	24/18	12/11	NA	69/73	15/15	11/10	12/18	0	63/65
IAMSSST	0	0	—	0	13/14	35/30	0	19/22	41/43	—	0	0
IAMCST	0	0	28/27	0	3/1	20/27	0	10/7	14/23	NA	0	0
FEVE, %	NA	NA	NA	NA	60/60	NA	60/60	58/58	NA	58/59	67/66	62/61
Doença multiarterial, %	60/65	62/60	NA	61/71	82/75	72/78	41/41	72/75	37/49	NA	0/0	0/0.8

Os valores são apresentados como médias, salvo indicação em contrário. O nível de significância estatística adotado nos estudos incluídos foi de 5%, quando disponível nas publicações originais. DAC: doença arterial coronariana; ECR: ensaio clínico randomizado; FEVE: fração de ejeção do ventrículo esquerdo; IAM: infarto agudo do miocárdio; IAMCST: IAM com supradesnivelamento do segmento ST; IAMSSST: IAM sem supradesnivelamento do segmento ST; NA: não aplicável.

**Tabela 2:** – Características angiográficas e procedimentais basais dos estudos incluídos

Estudo/Característica	Cortese, et al., 2010^21^	Latib, et al., 2012^22^	Sinaga, et al., 2016^27^	Giannini, et al., 2017^23^	Jeger, et al., 2018^28^	Sim, et al., 2018^24^	Tang, et al., 2018^29^	Cortese, et aI., 2020^31^	Silverio, et al., 2020^30^	Tsai, et al., 2022^26^	Kawai, et al., 2022^25^	Liu, et al., 2024^32^
Tamanho da amostra, n	28/29	90/92	172/163	90/91	382/376	87/200	116/110	118/114	1154/13634	47/59	19/23	129/118
*Follow-up* angiográfico, meses	6	24	12	12	12	12	9	6	36	12	8	9
Artéria descendente anterior esquerda, %	54/52	11/11	41/41	11/17	34/31	NA	10/10	39/40	49/45	42/44	42/17	22/23
Artéria diagonal, %	NA	17/8	NA	17/16	NA	NA	14/15	NA	NA	NA	NA	NA
Artéria circunflexa esquerda, %	18/10	11/16	32/34	11/13	47/49	NA	59/47	31/37	35/32	36/43	26/61	52/48
Tronco da coronária esquerda, %	NA	NA	NA	NA	NA	NA	NA	NA	0.4/0.5	NA	NA	NA
Artéria marginal obtusa, %	NA	27/32	NA	27/18	NA	NA	4/6	NA	NA	NA	NA	NA
Artéria coronária direita, %	28/38	8/9	26/23	8/11	20/20	NA	9/10	30/23	15/22	22/13	32/22	26/29
Artéria descendente posterior/posterolateral, %	NA	27/22	NA	27/20	NA	NA	20/26	NA	NA	NA	NA	NA
Comprimento da lesão, mm	12,4/11,3	15,4/14,4	NA	NA	NA	18,5/20,3	10,5/10,8	13,5/14	NA	19,8/20	11,8/11,3	12,3/11,5
Estenose do diâmetro, %	86/89	82/83	NA	82/82	NA	88/90	70/71	75/76	NA	87/86	68/60	69/71
Diâmetro mínimo do lúmen, mm	0,48/0,40	NA	NA	NA	NA	0,20/0,19	0,64/0,65	0,82/0,83	NA	0,29/0,29	0,75/0,80	0,68/0,65
Diâmetro de referência do vaso, mm	2,45/2,36	2,41/2,41	2,22/2,44	2,49/2,5	NA	1,88/1,95	2,42/2,42	2,23/2,18	2,23/2,18	2,1/2,1	2,29/2,08	2,2/2,21
Pressão do dispositivo, ATM	7,7/13,4	9,6/17,2	NA	9,6/17,8	11,1/13,6	NA	9,1/10,7	13,7/11,4	10/18	NA	7,7/13	10,2/10,8
Diâmetro do dispositivo, mm	2,48/2,54	2,49/2,49	2,25/2,5	2,49/2,5	2,75/2,57	NA	2,41/2,41	NA	2,5/2,5	2,09/2,18	2,6/2,4	2,44/2,42
Comprimento do dispositivo, mm	18,6/18,9	25,6/18,5	20,2/22,2	25,6/27,1	23,9/23,1	NA	21/20,4	21,8/18,3	20/18	26/25	19/17	20,5/19,4

Os valores são apresentados como números absolutos (n) ou percentuais (%). O nível de significância estatística adotado nos estudos incluídos foi de 5%, quando disponível nas publicações originais. ATM: atmosferas.

### Desfechos

**Figura 2:**
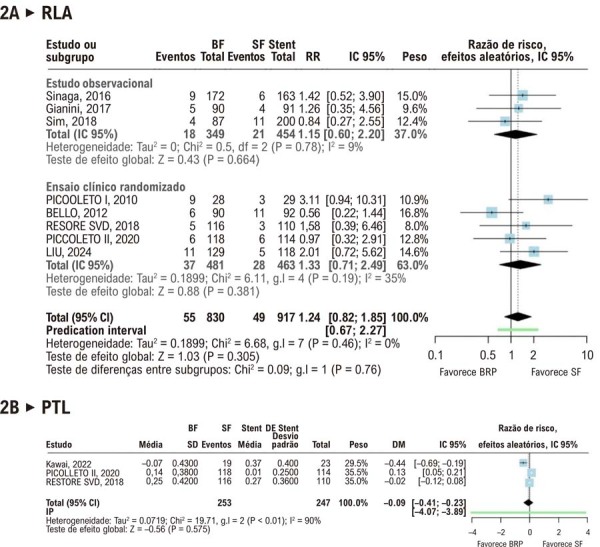
– Comparação da Revascularização da Lesão-Alvo e da Perda Tardia do Lúmen entre Balões Revestidos com Paclitaxel e Stents Liberadores de Fármacos em Doença de Pequenos Vasos. BF: balão farmacológico; BRP: balão revestido com paclitaxel; DM: diferença média; g.l.: graus de liberdade; IC 95%: intervalo de confiança de 95%; IP: intervalo de predição; PTL: perda tardia de lúmen; RLA: revascularização da lesão-alvo; RR: razão de risco; SF: stent farmacológico.

Em um *follow-up* médio de 32,4 meses, não foram observadas diferenças estatisticamente significativas entre os BFs e os SFs para nenhum dos desfechos analisados. O desfecho primário, RLA, ocorreu em 6,6% dos pacientes do grupo BF em comparação a 5,3% no grupo SF (RR, 1,24; IC 95%, 0,82-1,85; Figura 2A ). O desfecho angiográfico secundário, PTL, também foi semelhante entre os grupos (DM, –0,09 mm; IC 95%, –0,41 a 0,23), com valores médios de 0,17 mm no grupo BF e 0,16 mm no grupo SF ( Figura 2B ). Outros desfechos angiográficos não apresentaram diferenças significativas e estão descritos nas Figuras Suplementares S7A e S7B .

**Figura 3:**
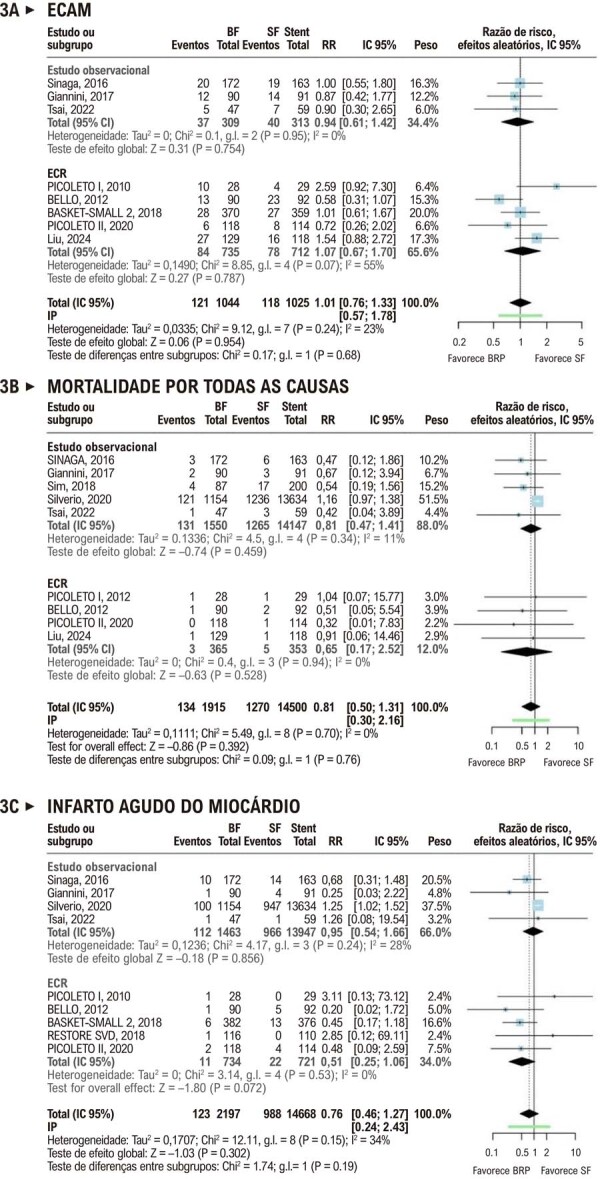
– Comparação de Eventos Cardiovasculares Adversos Maiores, Mortalidade por Todas as Causas e Infarto do Miocárdio entre Balões Revestidos com Paclitaxel e Stents Liberadores de Fármacos em Doença de Pequenos Vasos. BF: balão farmacológico; BRP: balão revestido com paclitaxel; DM: diferença média; ECAM: eventos cardiovasculares adversos maiores; ECR: ensaio clínico randomizado; g.l.: graus de liberdade; IC 95%: intervalo de confiança de 95%; IP: intervalo de predição; PTL: perda tardia de lúmen; RLA: revascularização da lesão-alvo; RR: razão de risco; SF: stent farmacológico.

Os desfechos clínicos também não diferiram significativamente entre os grupos. A RR para ECAM foi de 1,01 (IC 95%, 0,76-1,33; Figura 3A ); para mortalidade por todas as causas, 0,81 (IC 95%, 0,50-1,30; Figura 3B ); para mortalidade CV, 1,74 (IC 95%, 0,78-3,89; p = 0,17; I^2^ = 0%; PI = 0,01-323,77; Figura S8 ); e para IAM, 0,76 (IC 95%, 0,46-1,27; Figura 3C ). A Ilustração Central resume os principais achados da revisão.

As análises de subgrupos por delineamento dos estudos (ECRs vs. estudos de coorte não randomizados) apresentaram resultados consistentes.

### Avaliação da qualidade e certeza da evidência

O risco de viés nos ECRs e nos estudos de coorte não randomizados incluídos nesta metanálise foi considerado baixo ( Tabela S4 ).

**Figura 4:**
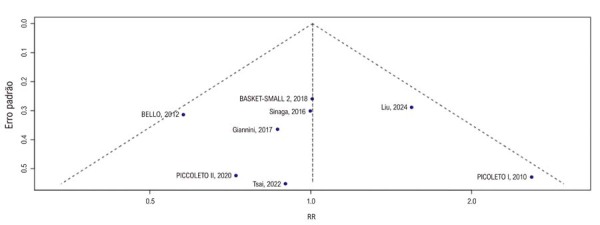
– Análise de gráfico em funil do desfecho de eventos cardiovasculares adversos maiores. RR: razão de risco.

A análise gráfica em funil do desfecho ECAM, utilizada para avaliar viés de publicação por inspeção visual de assimetria, sugeriu um viés favorável aos benefícios dos BF ( Figura 4 ). No entanto, o teste de Egger não apresentou significância estatística para viés de publicação. O GRADE encontra-se no Material Suplementar (Tabela S5) .

## Discussão

Esta revisão sistemática e metanálise que comparou BRP e SF na DACPV incluiu 12 estudos, abrangendo 17.441 pacientes, com *follow-up* variando de 8 a 36 meses (média de 32,4 meses). Não foram observadas diferenças significativas no desfecho primário, RLA motivada por critérios clínicos, nem nos desfechos secundários, como PTL, ECAM, mortalidade por todas as causas, mortalidade CV e IAM. Esses achados sustentam a segurança e eficácia dos BRP como alternativa viável aos SF no tratamento da DACPV. A consistência dos resultados entre os estudos, inclusive aqueles com BF de nova geração, reforça ainda mais essa evidência.

Nossa análise agrupada destacou uma alta prevalência de fatores de risco CV, com mais da metade dos pacientes apresentando hipertensão e mais de um terço com diabetes, refletindo uma população real submetida à ICP para DACPV.

O maior estudo incluído, o BASKET-SMALL 2 (Basel Stent Kosten Effektivitäts Trial Drug Eluting Balloons vs Drug Eluting Stents in Small Vessel Interventions),^28^ incluiu pacientes com pré-dilatação bem-sucedida da lesão e não encontrou diferença significativa em ECAM aos 12 meses entre os grupos BF e SF (7,3% vs. 7,5%; *hazard ratio* , 0,97; IC 95%, 0,58-1,64; *p* = 0,92), bem como taxas semelhantes de morte cardíaca, IAM e revascularização do vaso-alvo. Em contraste, o estudo PICCOLETO II (Drug Eluting Balloon Efficacy for Small Coronary Vessel Disease Treatment)^31^ demonstrou superioridade do BF sobre o SF quanto à PTL na lesão, aos 6 meses. O *follow-up* prolongado mostrou taxas semelhantes de mortalidade e IAM, porém maiores taxas de ECAM e oclusão aguda do vaso no grupo SF (20,8% vs. 10,8%, *p* = 0,046; e 4% vs. 0%, *p* = 0,042, respectivamente).^33^ Já o estudo REC-CAGEFREE I (Drug-Coated Balloon Angioplasty With Rescue Stenting Versus Intended Stenting for the Treatment of Patients With de Novo Coronary Artery Lesions)^34^ não estabeleceu a não inferioridade geral dos BRP; no entanto, os desfechos no subgrupo com DACPV (diâmetro do dispositivo < 3,0 mm) foram comparáveis ( *p* de interação = 0,02).

Embora as diretrizes atuais recomendem SF em vez de BF para reestenose intra- *stent* ,^35^ o uso de BF para lesões de novo em pequenos vasos tem ganhado espaço na prática clínica, especialmente entre pacientes com alto risco de sangramento. Embora ainda não esteja incorporada nas principais diretrizes, essa abordagem é apoiada por diversos consensos de especialistas.^8 - 10^ Uma vantagem importante do BF é a possibilidade de TAD mais curta, com duração de apenas 30 dias, reduzindo o risco de sangramento^36^ sem aumento na incidência de ECAM.^37^ Além disso, a ausência de implante permanente pode diminuir a ocorrência de trombose aguda e subaguda. Ainda assim, são necessários mais dados para confirmar a segurança e eficácia em longo prazo.

Os achados referentes ao risco de sangramento permanecem inconsistentes. Jeger et al.^28^ relataram um aumento de 2 vezes no risco de sangramento com o uso de SF em 1 ano, enquanto Cortese et al.^21^ não observaram diferenças significativas entre os dispositivos. No estudo DEBUT, que investigou pacientes com alto risco de sangramento tratados com apenas 1 mês de TAD, os BF foram associados a menores taxas de morte CV e IAM, embora as taxas de sangramento tenham sido semelhantes aos 9 meses.^38^ Importante destacar que os desfechos de sangramento foram reportados de forma inconsistente entre os estudos incluídos nesta metanálise, o que impossibilitou a realização de análise agrupada.

A reestenose continua sendo uma preocupação com o uso de BF. Uma análise com ajuste por escore de propensão do estudo SCAAR ( *Swedish Coronary Angiography and Angioplasty Registry* ) identificou maiores taxas de reestenose com os BF; no entanto, não foram observadas diferenças em desfechos duros, como trombose da lesão-alvo, IAM ou óbito.^30^ De forma semelhante, Latib et al.^22^ relataram desfechos comparáveis em um ECR com *follow-up* de 2 anos. Devido à inconsistência na forma de relato, a reestenose não foi analisada nesta metanálise.

Os BFs também podem ser utilizados como parte de uma estratégia híbrida, especialmente em lesões longas ou difusas. Nessa abordagem, os SF são geralmente implantados em vasos de maior calibre com doença residual após a angioplastia com balão, enquanto os BF são reservados para vasos de menor calibre, mais suscetíveis à reestenose, disfunção vasomotora, neoaterosclerose e maiores desafios em cirurgias de revascularização do miocárdio.^1 , 39^

Embora todos os BRP liberem paclitaxel, eles diferem em formulação e características do dispositivo, como o excipiente (p.ex., iopromida [SeQuent^®^ Please, B. Braun] vs. ureia [IN.PACT™ Falcon™, Medtronic]) e o perfil do balão. Excipientes hidrofílicos como a ureia podem facilitar a transferência do fármaco e podem ser vantajosos em lesões curtas e simples.^40^ Flexibilidade e navegabilidade dos dispositivos também variam, o que pode influenciar no sucesso do procedimento em anatomias complexas.

Esta metanálise apresenta várias limitações. A maioria dos estudos incluídos não realizou *follow-up* angiográfico, o que restringiu a análise de PTL e reestenose. Como consequência, o estudo de Silverio et al.^30^ — o maior desta metanálise — foi excluído da análise de RLA por ausência de dados disponíveis. Além disso, 11 dos 12 estudos utilizaram SF de segunda geração, fator que deve ser considerado na extrapolação dos resultados.^21^ Outra limitação é a ausência de uma definição universalmente aceita para DACPV, uma vez que os critérios de diâmetro variaram entre os estudos. Por fim, o número limitado de ECRs exigiu a inclusão de estudos de coorte não randomizados. Embora os ECRs apresentem maior validade interna, os dados observacionais fornecem informações valiosas sobre desfechos em longo prazo.

## Conclusão

Esta revisão sistemática e metanálise, que incluiu 12 estudos, não identificou diferença estatisticamente significativa entre os BRP e os SF no tratamento da DACPV. Esses achados sustentam o potencial dos BF como alternativa segura e eficaz aos *stents* no manejo da DACPV. No entanto, são necessários novos ECRs com *follow-ups* mais prolongados para confirmar esses resultados e orientar sua adoção mais ampla na prática clínica rotineira, especialmente para lesões de novo em pequenos vasos coronarianos.

## *Material suplementar

SUPPLEMENTAL MATERIAL
