# Supplementary material for: Balões Revestidos com Paclitaxel versus Stents Farmacológicos na Doença Arterial Coronariana de Pequenos Vasos: Revisão Sistemática e Metanálise
Source: Arq Bras Cardiol. 2025 Nov 26;122(10):e20250069. [Article in Portuguese] doi: 10.36660/abc.20250069 (PMC12711004; doi:10.36660/abc.20250069)
Supplement: SUPPLEMENTAL MATERIAL [file 0066-782x-abc-122-10-e20250069-suppl01.pdf]

SUPPLEMENTAL MATERIAL

Search strings.

Table S1. Baseline characteristics of individual studies.

Figure S1: Risk of bias assessment using Newcastle-Ottawa Score of the studies assessed as case-control.

Figure S2: Risk of bias assessment using QUADAS-2 of the studies assessed as diagnostic test accuracy.

Figure S3: SROC plot, 95% confidence region and 95% predictive region of the comparison between new or indeterminate versus old LBBB.

Figure S4: SROC plot, 95% confidence region and 95% predictive region of the comparison between new versus indeterminate or old LBBB.

Figure S5: Sensitivity and specificity forest plots for Sgarbossa criteria.

Figure S6: Sensitivity analysis of Sgarbossa criteria including only studies with angiographically defined MI and excluding pacemaker's studies.

Figure S7: SROC plot, 95% confidence region and 95% predictive region of Sgarbossa Criteria including studies with angiographically defined MI.

Figure S8: Sensitivity and specificity forest plots for Modified Sgarbossa criteria.

Figure S9: Sensitivity analysis of Modified Sgarbossa criteria including only studies with angiographically defined MI and excluding pacemaker's studies.

Figure S10: SROC plot, 95% confidence region and 95% predictive region of Sgarbossa Modified Criteria including studies with angiographically defined MI.

Figure S11: Sensitivity and specificity forest plots for Barcelona criteria.

Figure S12: Sensitivity and specificity forest plots for Chapman sign.

Figure S13: Funnel plot, Egger's and Begg's test P values of the analysis examining the incidence and prevalence of left bundle branch block (LBBB) in acute coronary syndromes (ACS).

Figure S14: Funnel plot, Egger's and Begg's test P values of the analysis examining the in-hospital mortality of left bundle branch block (LBBB) compared to right bundle branch block in acute coronary syndromes (ACS).

Figure S15: Funnel plot, Egger's and Begg's test P values of the analysis examining the in-hospital mortality of left bundle branch block (LBBB) compared to STEMI in acute coronary syndromes (ACS).

Figure S16: Funnel plot, Egger's and Begg's test P values of the analysis examining the in-hospital mortality of left bundle branch block (LBBB) compared to patients without bundle branch blocks in acute coronary syndromes (ACS).

Search strings

Pubmed – 2023/12/21

("left bundle branch block" OR "LBBB") AND ("acute coronary occlusion" OR "coronary artery occlusion" OR "myocardial infarction" OR "ST-segment elevation myocardial infarction" OR "STEMI" OR "acute coronary syndrome" OR "primary percutaneous coronary intervention" OR "PCI") NOT ("Review" [Publication Type] OR "Case Reports" [Publication Type])

Scopus – 2023/12/21

TITLE-ABS-KEY(("left bundle branch block" OR "LBBB") AND ("acute coronary occlusion" OR "coronary artery occlusion" OR "myocardial infarction" OR "ST-segment elevation myocardial infarction" OR "STEMI" OR "acute coronary syndrome" OR "primary percutaneous coronary intervention" OR "PCI")) AND (DOCTYPE(ar)) AND NOT (DOCTYPE(re) OR DOCTYPE(cr) OR DOCTYPE(cp) OR DOCTYPE(bs) OR DOCTYPE(ch) OR DOCTYPE(no))

Table S1.

Study	Study design	Included patients	Sample Size	Outcomes of interest	Reference standards
Go 1998	Retrospective cohort	Acute myocardial infarction with left, right or no bundle branch block	297832	In-hospital mortality, prevalence	CKMB
Brilakis 2001	Retrospective cohort	Acute myocardial infarction with left, right or no bundle branch block	894	In-hospital mortality	Unspecified biomarkers
Yeo 2011	Retrospective cohort	STEMI or new left bundle branch block	46006	In-hospital mortality	Universal definition of MI
Knot 2012	Retrospective cohort	Patients with AMI regardless of ECG finding	6602	In-hospital mortality	Universal definition of MI
Guerrero 2005	Post hoc analysis of a clinical trial	Clinically diagnosed STEMI or new LBBB	3053	In-hospital mortality	Universal definition of MI
Lopes 2011	Post hoc analysis of a clinical trial	Clinically diagnosed STEMI or new LBBB	5742	In-hospital mortality, prevalence	Universal definition of MI
Meyer 2020	Retrospective cohort	Acute myocardial infarction	33157	In-hospital mortality, prevalence	Troponin
Jones 1977	Retrospective cohort	Acute myocardial infarction	556	In-hospital mortality, prevalence	AST/ALT
Moreno 2002	Retrospective cohort	Clinically diagnosed STEMI or new LBBB	945	In-hospital mortality, prevalence	Universal definition of MI
Chang 2009	Retrospective cohort	Patients with chest pain	7937	Prevalence	Universal definition of MI
Erne 2017	Retrospective cohort	Clinically diagnosed STEMI or new LBBB	29114	In-hospital mortality	Universal definition of MI
Timoteo 2018	Retrospective cohort	Acute myocardial infarction	3990	In-hospital mortality, prevalence	Universal definition of MI
Vivas 2010	Retrospective cohort	Clinically diagnosed STEMI or new LBBB	913	In-hospital mortality, prevalence	Universal definition of MI
Lewinter 2011	Post hoc analysis of a clinical trial	Clinically diagnosed STEMI or new LBBB	6676	In-hospital mortality, prevalence	Universal definition of MI
Moreno 1999	Retrospective cohort	Acute myocardial infarction	1239	In-hospital mortality	CPK
Al-Faleh 2006	Post hoc analysis of a clinical trial	Clinically diagnosed STEMI or new LBBB	22839	Prevalence	Universal definition of MI
Di Marco 2016	Retrospective cohort	Clinically diagnosed STEMI or new LBBB	251	Prevalence	Universal definition of MI

Mozid 2015	Retrospective cohort	Clinically diagnosed STEMI or new LBBB	1875	Prevalence, diagnostic test accuracy	Acute coronary occlusion
Nestelberger 2019	Retrospective cohort	Patients with chest pain	8830	Prevalence	Universal definition of MI
Turnipseed 2009	Retrospective cohort	Patients with chest pain	340	Prevalence	Universal definition of MI
Steinmetz 1979	Retrospective cohort	Acute myocardial infarction	404	Prevalence	AST/ALT
Alkindi 2014	Retrospective cohort	Acute myocardial infarction	50992	Prevalence	Unspecified biomarkers
van der Ende 2017	Retrospective cohort	Clinically diagnosed STEMI or new LBBB	1123	Prevalence	Universal definition of MI
Pera 2018	Retrospective cohort	Clinically diagnosed STEMI or new LBBB	3903	Prevalence	Universal definition of MI
Kontos 2011	Retrospective cohort	Chest pain in LBBB patients	401	Risk of myocardial infarction	Universal definition of MI
Mehta 2012	Retrospective cohort	Clinically diagnosed STEMI or new LBBB	802	Prevalence, risk of myocardial infarction	Universal definition of MI
Flora 2020	Retrospective cohort	Clinically diagnosed STEMI or new LBBB	357	Prevalence	Universal definition of MI
Edhouse 1999	Retrospective cohort	Patients with chest pain	797	Prevalence, diagnostic test accuracy	CPK
Shojaeefard 2022	Cross-sectional	Acute myocardial infarction	5233	Prevalence	Universal definition of MI and Modified Sgarbossa Criteria
Col 1972	Retrospective cohort	Acute myocardial infarction	208	Prevalence	Q waves
Archbold 2010	Retrospective cohort	Patients with chest pain	3890	Prevalence	CPK
Lai 2020	Retrospective cohort	Clinically diagnosed STEMI or new LBBB	2432	Prevalence, diagnostic test accuracy	Universal definition of MI
McMahon 2013	Retrospective cohort	Patients with chest pain	1454	Prevalence, diagnostic test accuracy	Universal definition of MI
Wong 2005	Post hoc analysis of a clinical trial	Clinically diagnosed STEMI or new LBBB	17073	Prevalence, risk of myocardial infarction	Universal definition of MI
Tolppanen 2018	Retrospective and prospective cohort	Cardiogenic shock complicating AMI	199	Prevalence	Universal definition of MI

Kontos 2001	Cross-sectional	Patients with chest pain	7725	Diagnostic test accuracy	CKMB
Shlipak 2000	Case-control	Acute myocardial infarction with left bundle branch block	94	Diagnostic test accuracy	Troponin or CKMB
Wegmann 2015	Cross-sectional	Clinically suspected STEMI or new LBBB	1139	Diagnostic test accuracy	Acute coronary occlusion
Li 2000	Retrospective cohort	Acute myocardial infarction with left bundle branch block	306	Diagnostic test accuracy	CKMB
Liakopoulos 2013	Retrospective cohort	Acute myocardial infarction with left bundle branch block	99	Diagnostic test accuracy	Troponin
Kontos 2011	Cross-sectional	Patients with chest pain	401	Diagnostic test accuracy	Troponin or CKMB
Lindow 2023	Retrospective cohort	Patients with chest pain	> 20000	Diagnostic test accuracy	Acute coronary occlusion
Smith 2012	Case-control	Patients with LBBB acute coronary occlusions for cases and patients with LBBB without acute coronary occlusions for controls	162	Diagnostic test accuracy	Acute coronary occlusion
Freitas 2016	Prospective study	Patients with chest pain and pacemakers	51	Diagnostic test accuracy	Acute coronary occlusion
Dodd 2021	Case-control	Patients with chest pain and pacemakers	176	Diagnostic test accuracy	Acute coronary occlusion
Sgarbossa 1996	Post hoc analysis of a clinical trial	Patients with LBBB and acute myocardial for cases; LBBB without acute myocardial infarctions for controls	145	Diagnostic test accuracy	Unspecified biomarkers
Sokolove 2000	Post hoc analysis of a clinical trial	Patients with LBBB and acute myocardial for cases; LBBB without acute myocardial infarctions for controls	224	Diagnostic test accuracy	CKMB
Meyers 2015	Case-control	Patients with chest pain and LBBB	294	Diagnostic test accuracy	Acute coronary occlusion

Di Marco 2020	Case-control	Clinically suspected STEMI or new LBBB for cases and patients with LBBB and no chest pain for controls	484	Diagnostic test accuracy	Troponin
Wackers 1983	Case-control	Patients with LBBB in coronary units for cases, and outpatients with LBBB for controls	96	Diagnostic test accuracy	Unspecified biomarkers
Hands 1988	Cross-sectional	Acute myocardial infarction	985	Diagnostic test accuracy	CKMB

CAPTION: Baseline characteristics of individual studies. Note – When a primary study reported hypothesis-testing, it consistently applied a two-sided significance level of 0.05; for purely descriptive prevalence or diagnostic-accuracy reports, no α level was stated or required.

Figure S1.

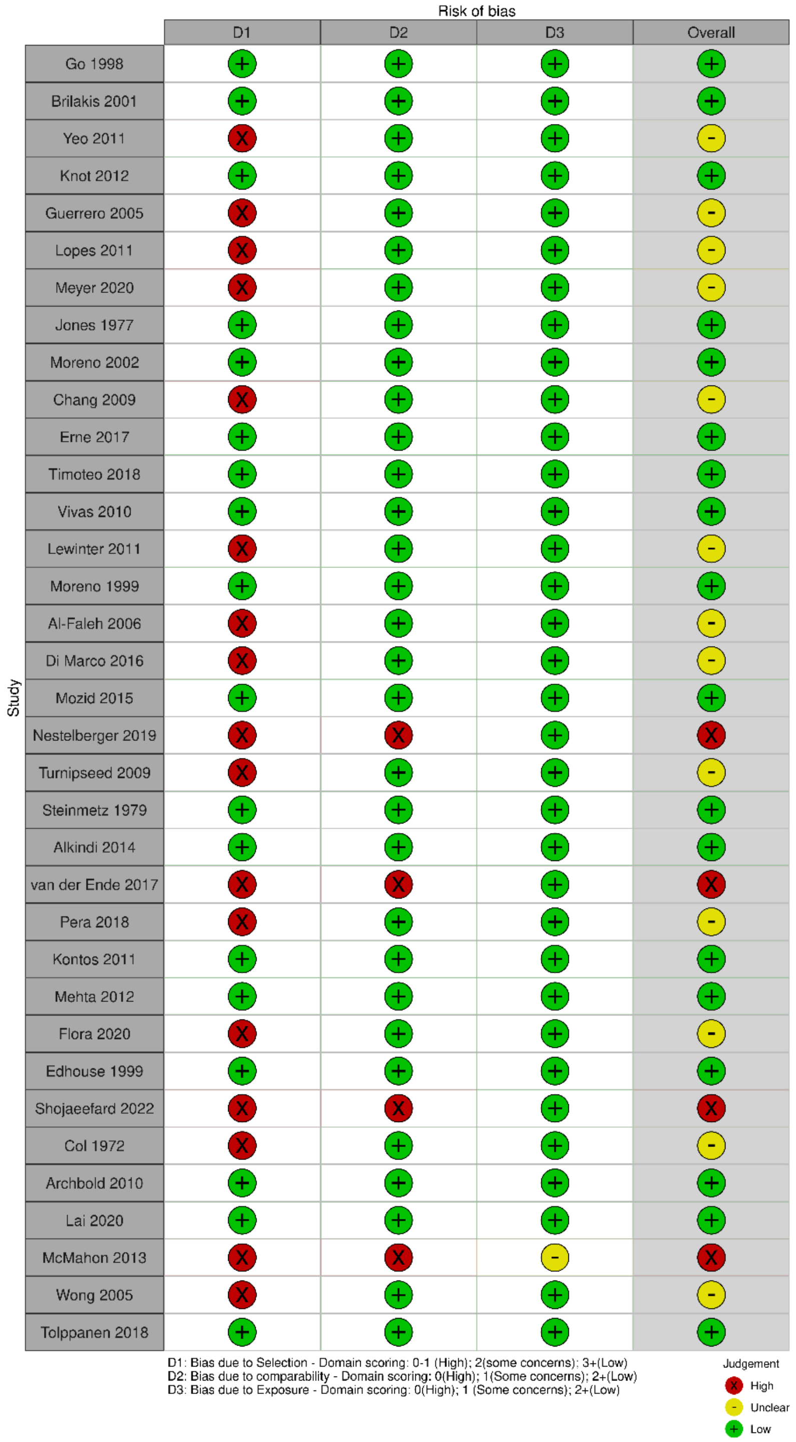

Caption: Risk of bias assessment using Newcastle-Ottawa Score of the studies assessed as case-control.

Figure S2.

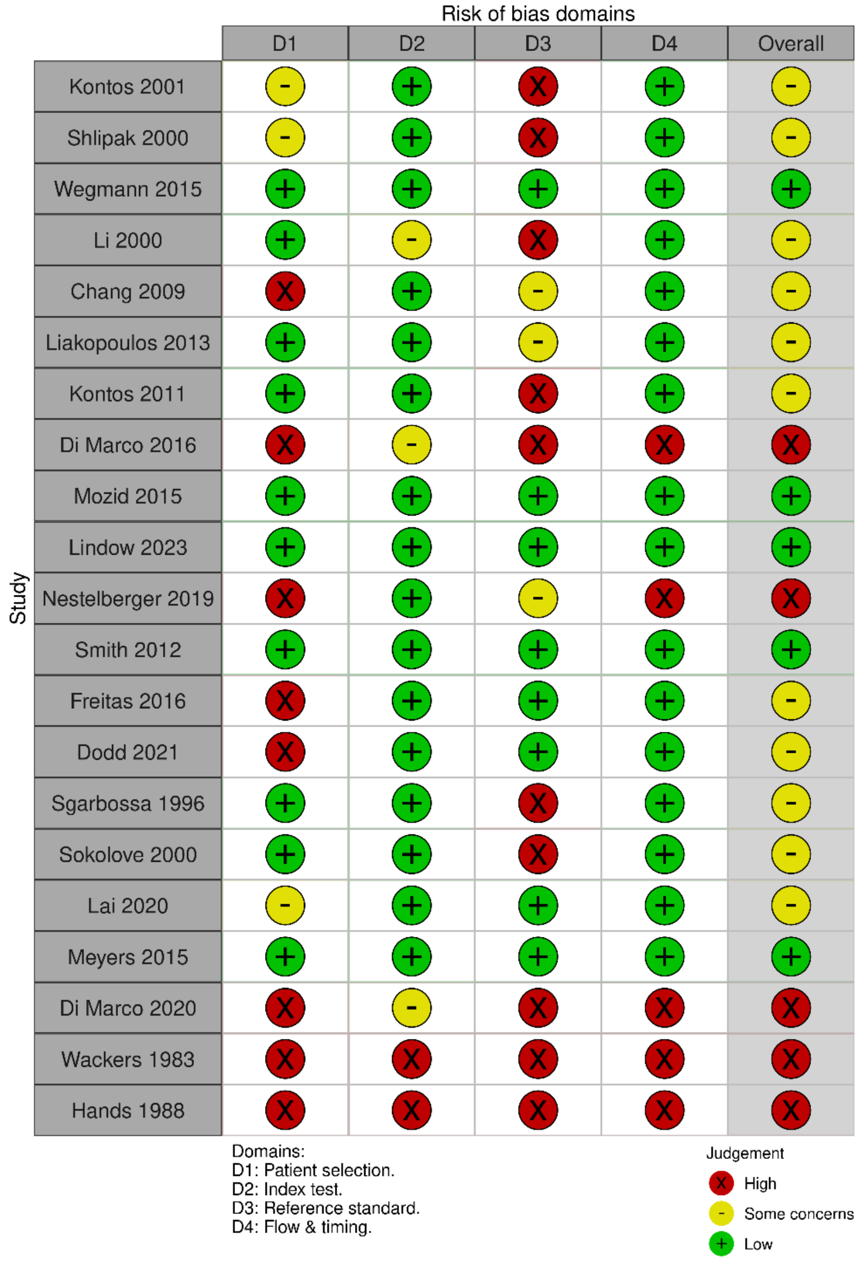

Caption: Risk of bias assessment using QUADAS-2 of the studies assessed as diagnostic test accuracy.

Figure S3.

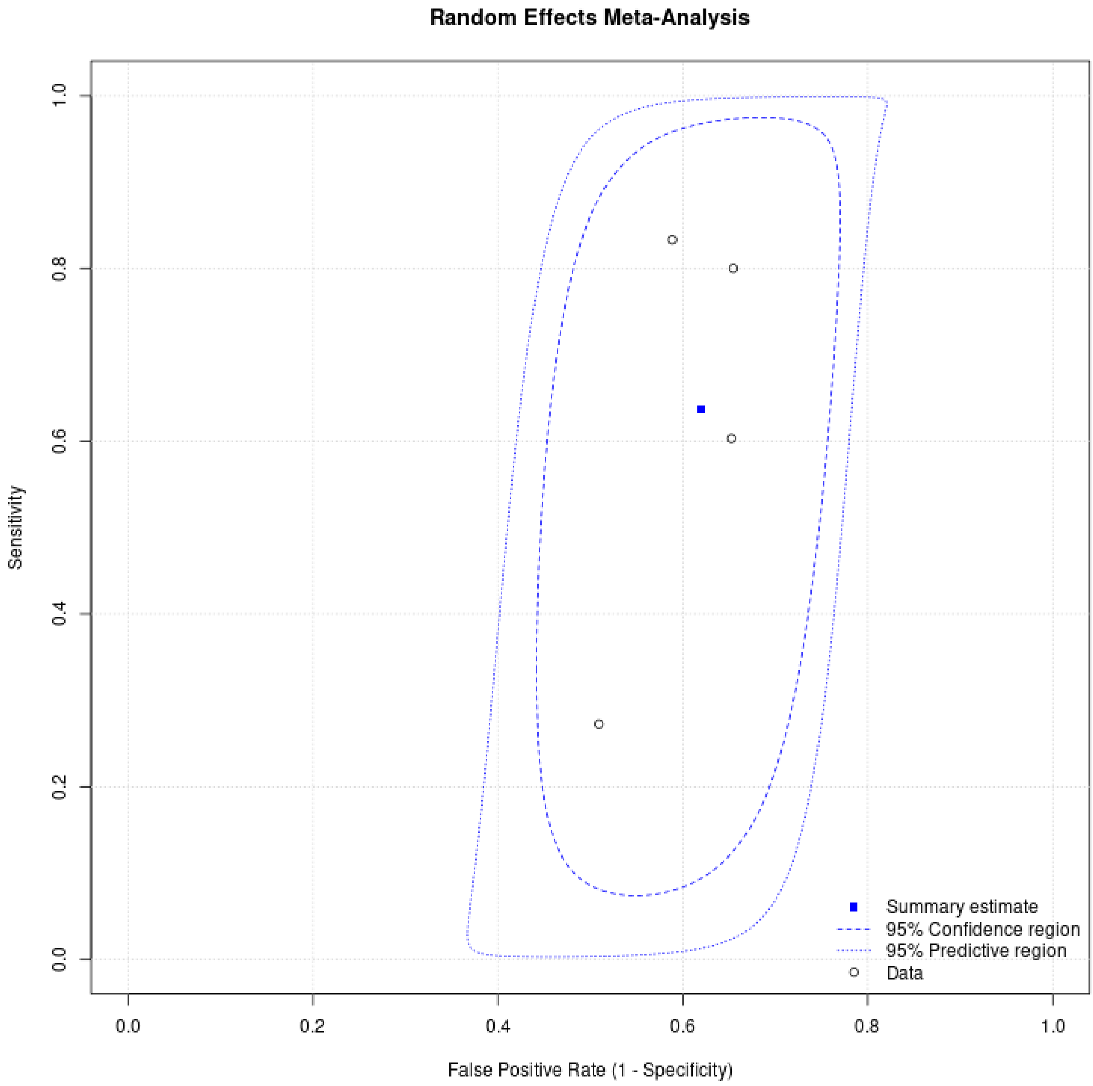

CAPTION: SROC plot, 95% confidence region and 95% predictive region of the comparison between new or indeterminate versus old LBBB.

Figure S4.

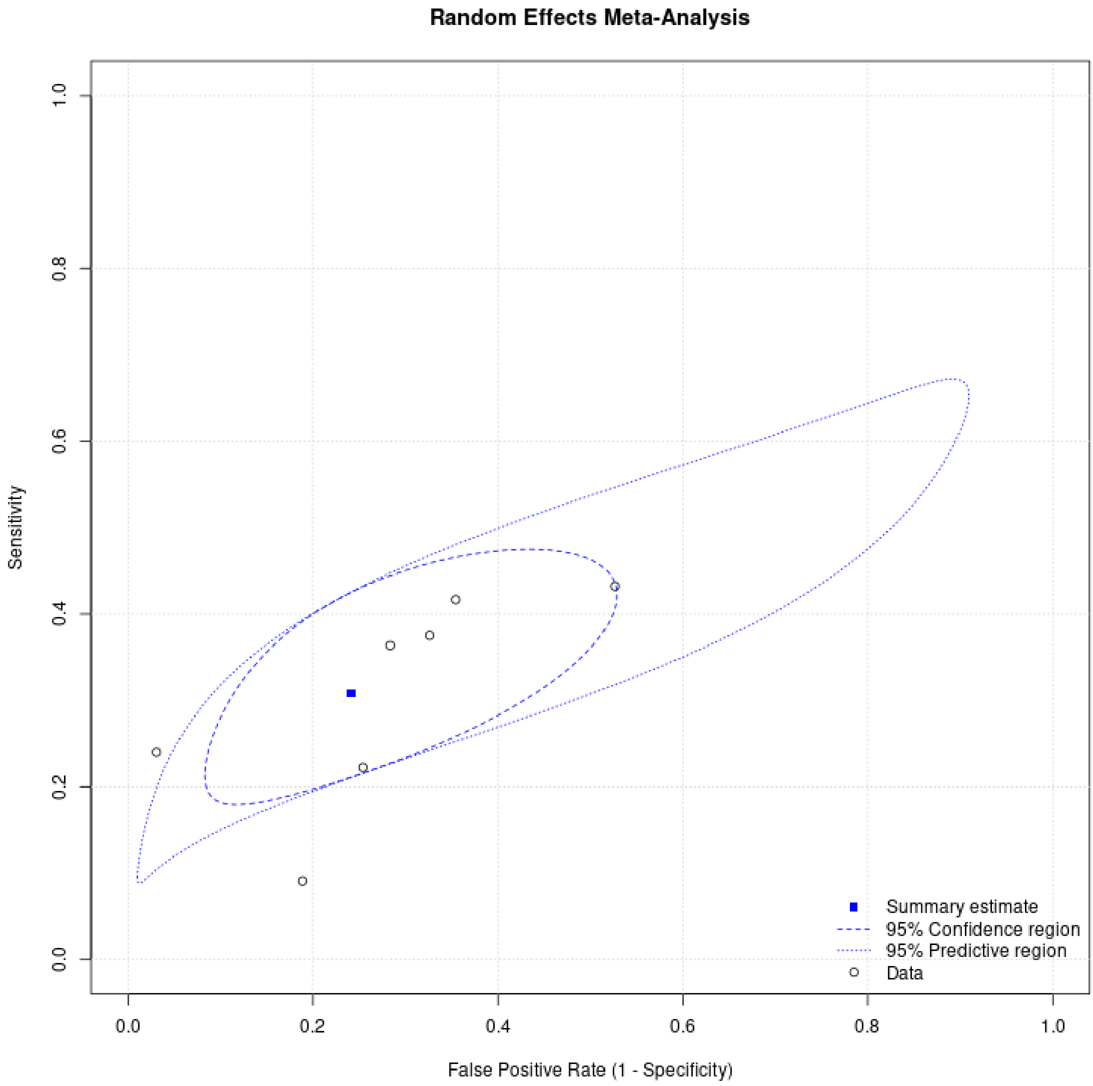

CAPTION: SROC plot, 95% confidence region and 95% predictive region of the comparison between new versus indeterminate or old LBBB.

Figure S5.

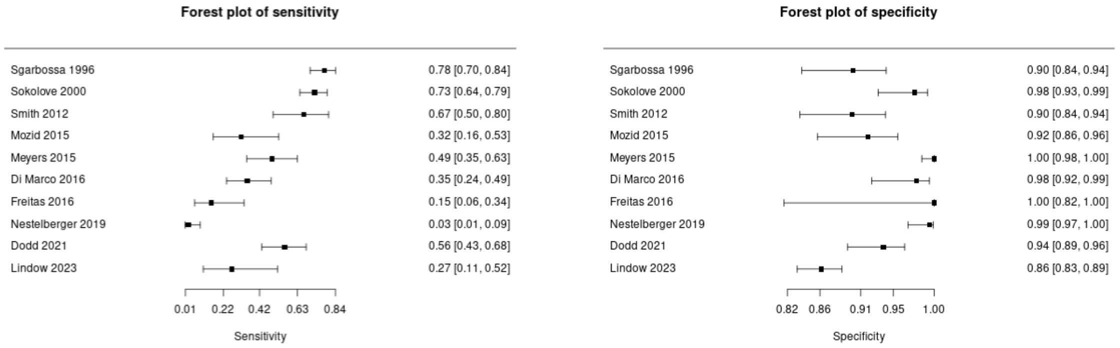

Caption. Sensitivity and specificity forest plots for Sgarbossa criteria.

Figure S6.

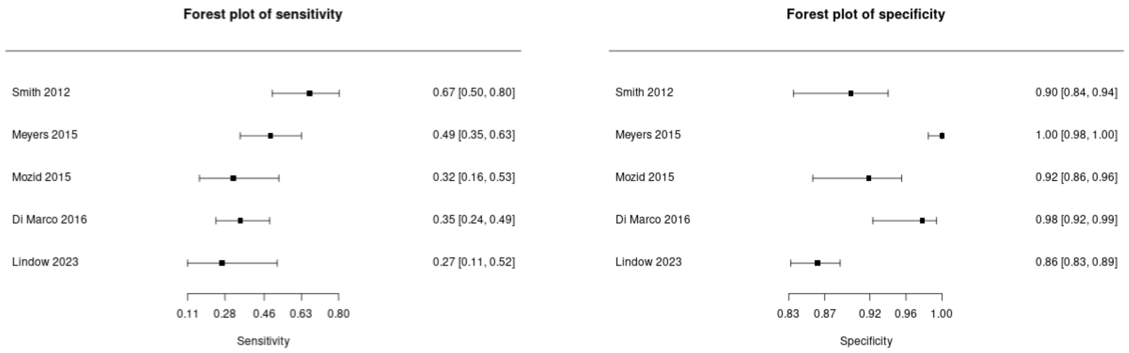

Caption. Sensitivity analysis of Sgarbossa criteria including only studies with angiographically defined MI and excluding pacemaker’s studies.

Figure S7.

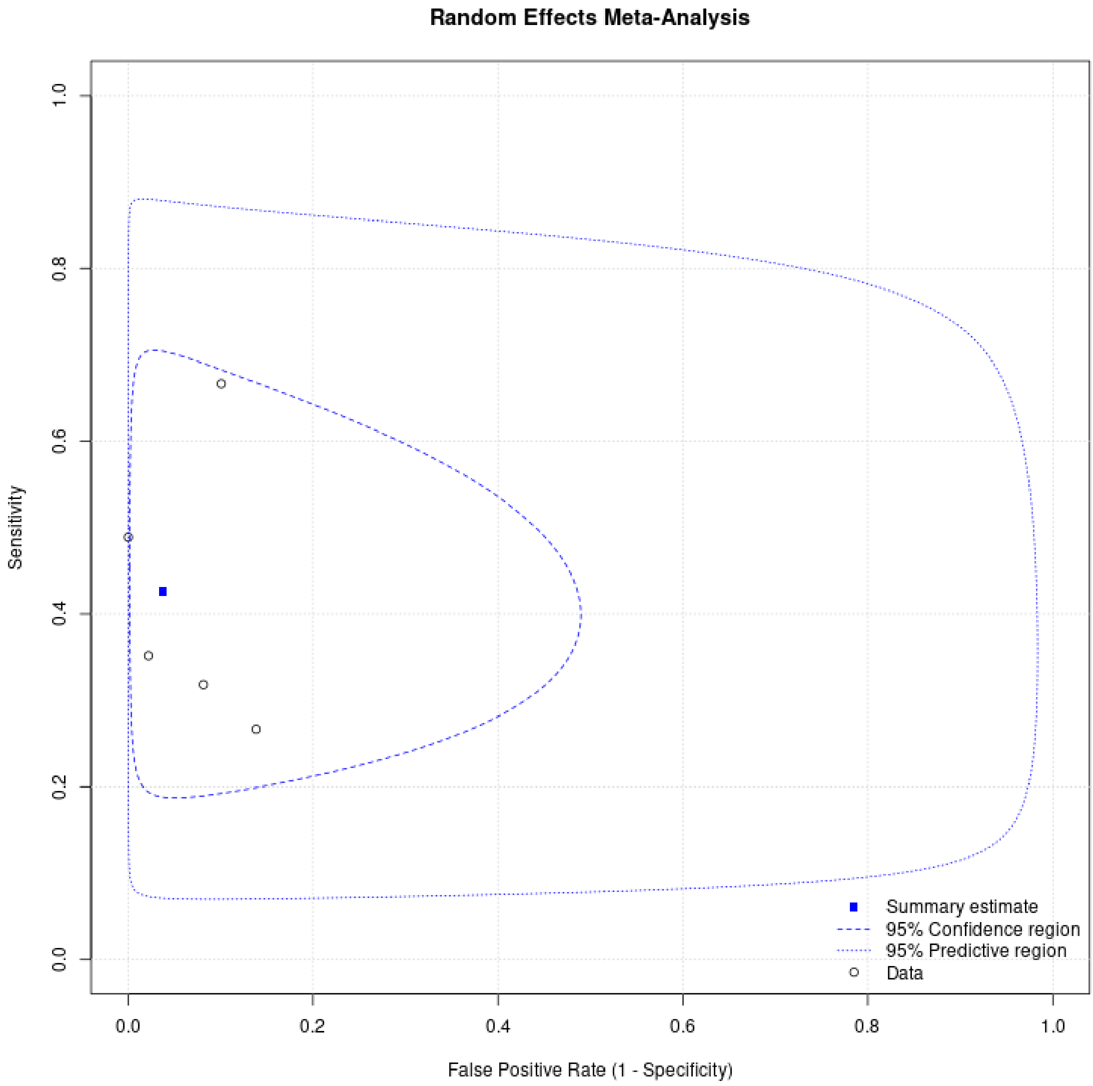

CAPTION. SROC plot, 95% confidence region and 95% predictive region of Sgarbossa Criteria including studies with angiographically defined MI.

Figure S8.

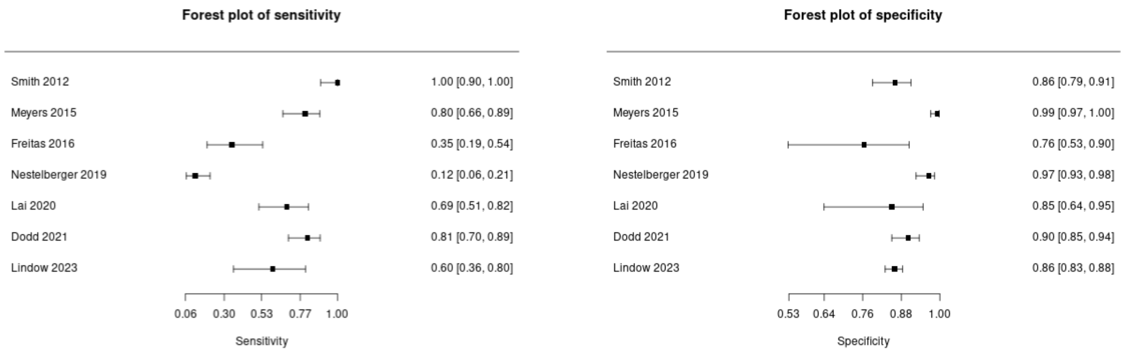

Caption. Sensitivity and specificity forest plots for Modified Sgarbossa criteria.

Figure S9.

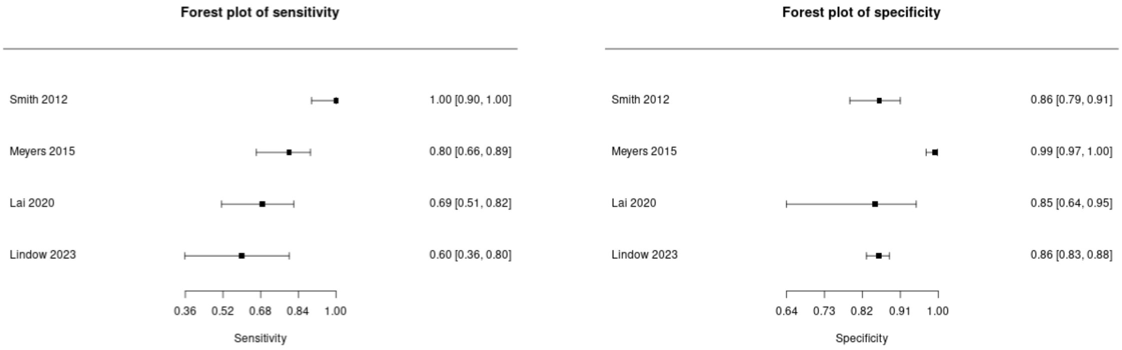

Caption. Sensitivity analysis of Modified Sgarbossa criteria including only studies with angiographically defined MI and excluding pacemaker's studies.

Figure S10.

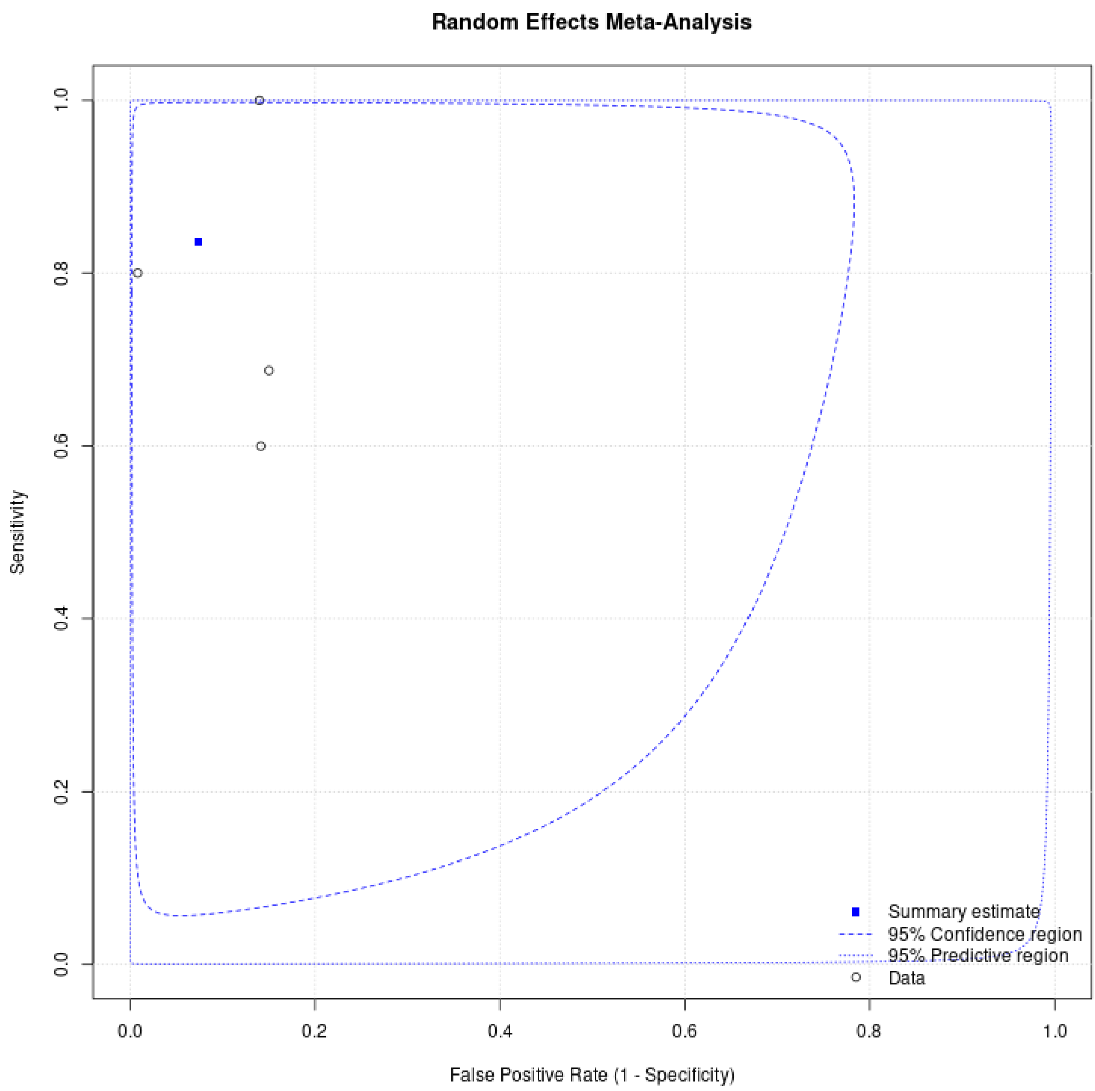

CAPTION. SROC plot, 95% confidence region and 95% predictive region of Sgarbossa Modified Criteria including studies with angiographically defined MI.

Figure S11.

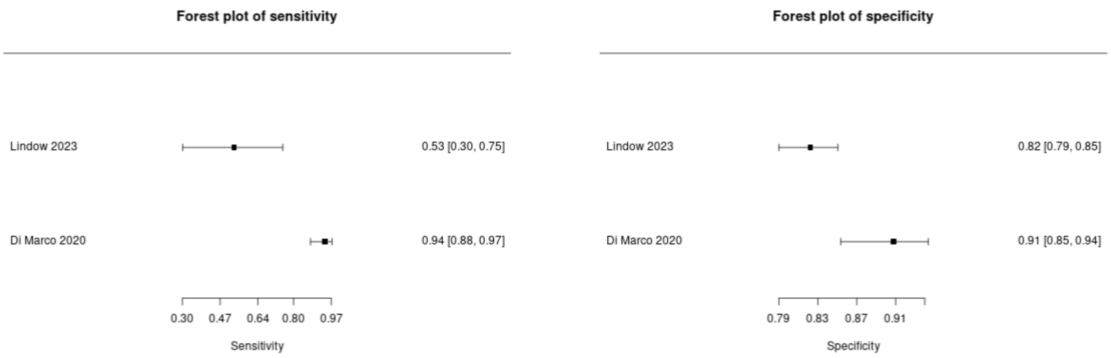

Caption. Sensitivity and specificity forest plots for Barcelona criteria.

Figure S12.

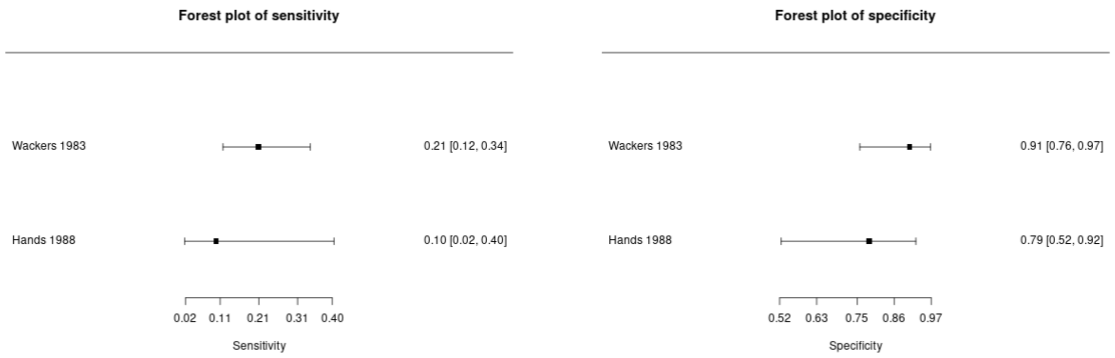

Caption. Sensitivity and specificity forest plots for Chapman sign.

Figure S13.

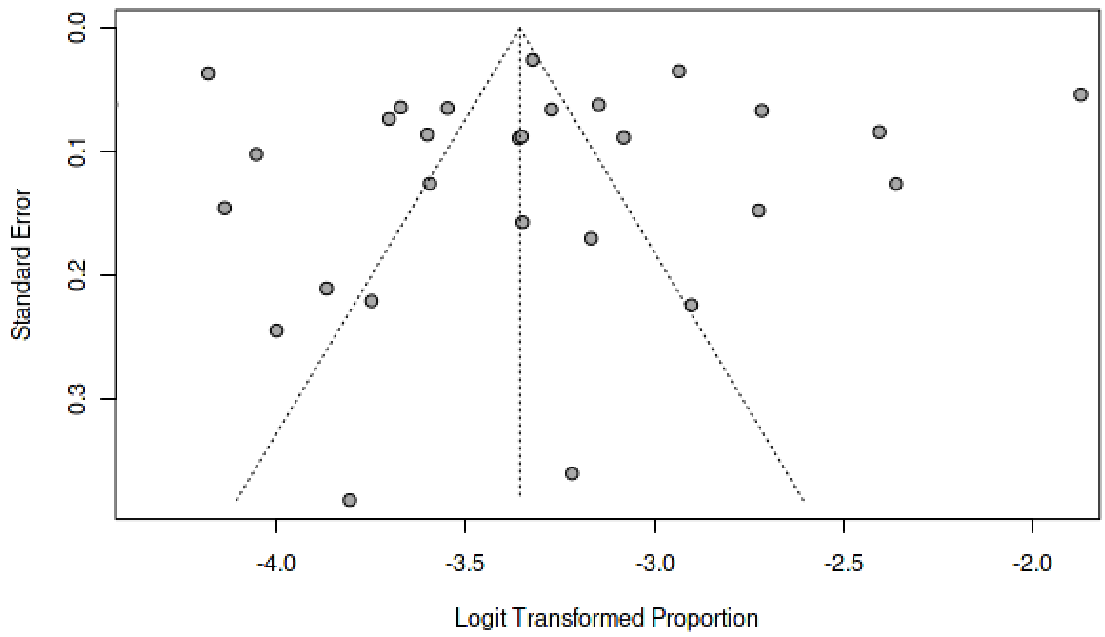

P value of Egger's test: 0.9953

P value of Begg's test: 0.8807

Caption: Funnel plot, Egger's and Begg's test P values of the analysis examining the incidence and prevalence of left bundle branch block (LBBB) in acute coronary syndromes (ACS).

Figure S14.

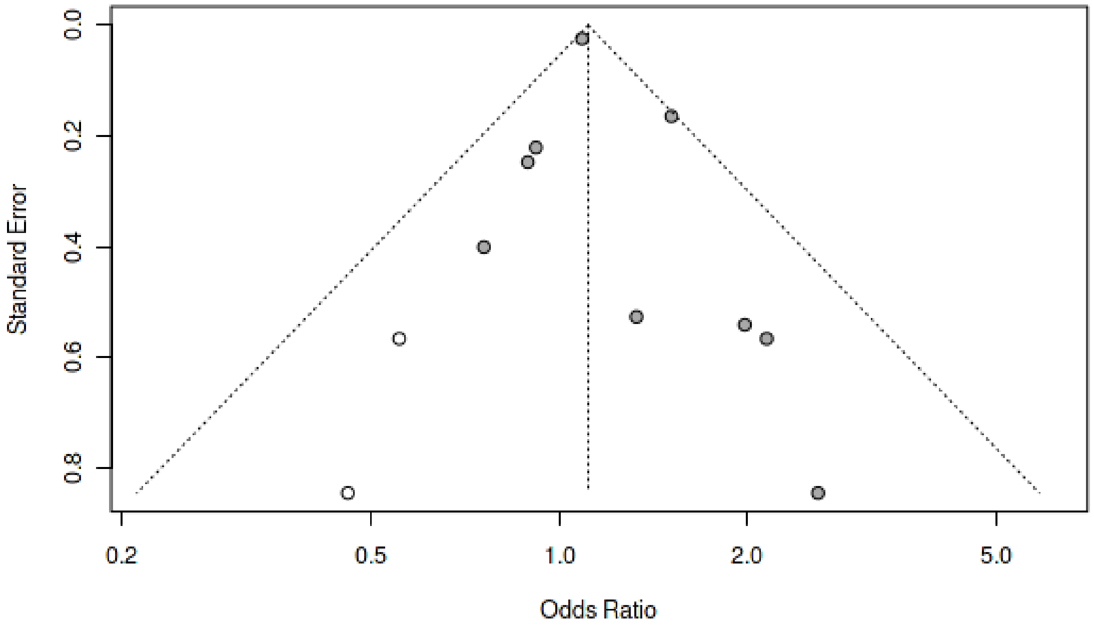

P value of Egger's test: 0.3438
P value of Begg's test: 0.2109

CAPTION. Funnel plot, Egger’s and Begg’s test P values of the analysis examining the in-hospital mortality of left bundle branch block (LBBB) compared to right bundle branch block in acute coronary syndromes (ACS).

Figure S15.

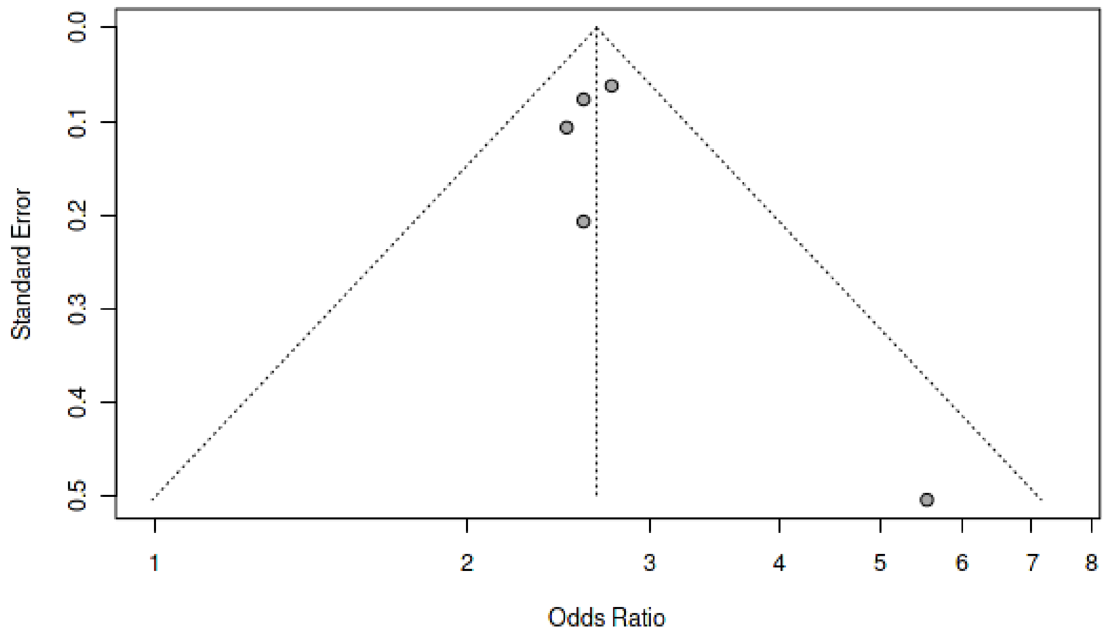

P value of Egger's test: 0.4565
P value of Begg's test: 0.6242

CAPTION. Funnel plot, Egger's and Begg's test P values of the analysis examining the in-hospital mortality of left bundle branch block (LBBB) compared to STEMI in acute coronary syndromes (ACS).

Figure S16.

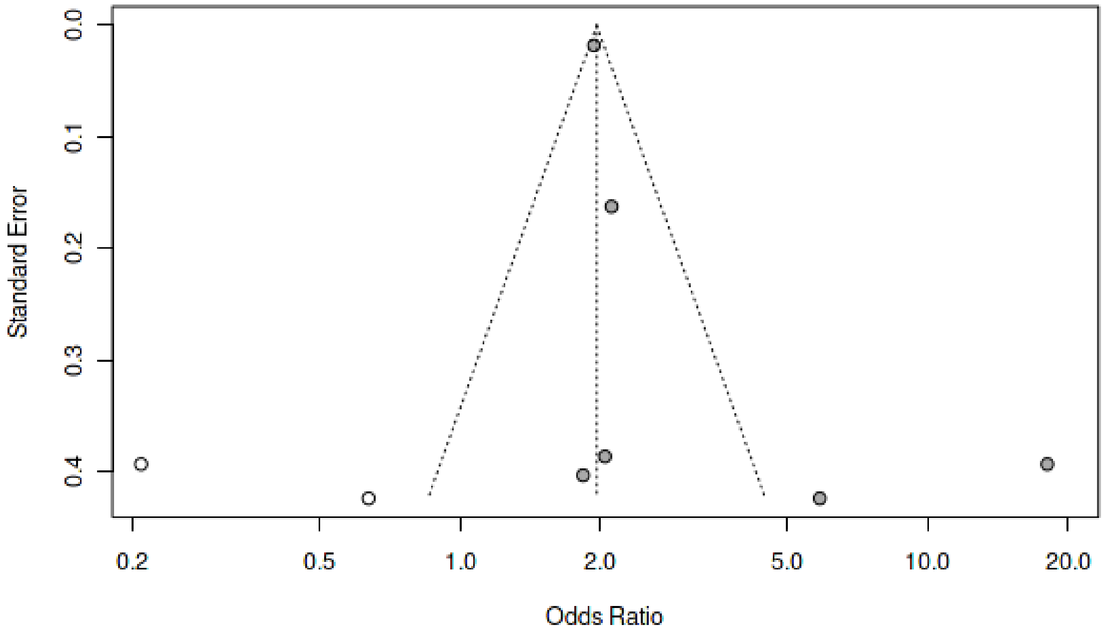

P value of Egger's test: 0.1769

P value of Begg's test: 0.3476

CAPTION. Funnel plot, Egger's and Begg's test P values of the analysis examining the in-hospital mortality of left bundle branch block (LBBB) compared to patients without bundle branch blocks in acute coronary syndromes (ACS).
