# Supplementary material for: Balões Revestidos com Paclitaxel versus Stents Farmacológicos na Doença Arterial Coronariana de Pequenos Vasos: Revisão Sistemática e Metanálise
Source: Arq Bras Cardiol. 2025 Nov 26;122(10):e20250069. [Article in Portuguese] doi: 10.36660/abc.20250069 (PMC12711004; doi:10.36660/abc.20250069)
Supplement: Translation

# Translation: Paclitaxel-Coated Balloons versus Drug-Eluting Stents in Small Vessel Coronary Artery Disease: A Systematic Review and Meta-Analysis

**DOI:** 10.36660/abc.20250069i

**Authors:** Marília Oberto da Silva Gobbo, Renan Yuji Ura Sudo, Ancy Jenil Franco, Mable Pereira, Tanize Louize Milbradt, Zeynep Eylul Bakir, Iago Zang Pires, Stephen Akinfenwa, Gustavo Luís Agostini, Denise Pellegrini, Paulo R. A. Caramori

**Affiliations:** 1 Brazil Pontifícia Universidade Católica do Rio Grande do Sul, Porto Alegre, RS – Brazil; 2 Brazil Universidade Federal da Grande Dourados, Dourados, MS – Brazil; 3 USA McLaren Bay Region, Internal Medicine Department, Bay City, Michigan – USA; 4 Guyana Lincoln American University School of Medicine, Georgetown – Guyana; 5 Brazil Universidade Federal de Santa Maria, Santa Maria, RS – Brazil; 6 Italy University of Rome La Sapienza, Rome, Lazio – Italy; 7 USA University of Connecticut, Storrs – USA; 8 Brazil Tacchini Hospital, Bento Gonçalves, RS – Brazil; 9 Brazil Hospital São Lucas da PUCRS, Porto Alegre, RS – Brazil

**Keywords:** Drug-Eluting Stents, Coronary Artery Disease, Percutaneous Coronary Intervention

## Abstract

**Background:**

Drug-eluting stents (DES) remain the standard of care for percutaneous coronary intervention in small vessel coronary artery disease (SVD). Paclitaxel-coated balloons (PCB) have emerged as an alternative, with recent trials suggesting favorable short- and mid-term outcomes, although most were limited by small sample sizes.

**Objectives:**

To compare angiographic and clinical outcomes between DES and PCB in patients with SVD.

**Methods:**

Searches were conducted in PubMed, Embase, the Cochrane Library, and ClinicalTrials.gov for studies comparing PCB and DES in SVD, defined as a reference vessel diameter < 3.0 mm. A random-effects meta-analysis was performed, and results were reported as mean differences (MD) or risk ratios (RR). Statistical significance was set at p < 0.05. Heterogeneity was assessed using the chi-square, Tau, and Tau^2^tests. The review protocol was registered in PROSPERO (CRD42024506502).

**Results:**

A total of 12 studies comprising 17,441 patients (mean age of 58-73 years) were included, with clinical follow-up of 8 to 36 months. All studies evaluated PCB. No significant differences were found between PCB and DES in target lesion revascularization (RR, 1.24; 95% CI, 0.82-1.85; p = 0.30), late lumen loss (MD, –0.09 mm; 95% CI, –0.41 to 0.23; p = 0.57), major adverse cardiovascular events (RR, 1.01; 95% CI, 0.76-1.33; p = 0.95), all-cause mortality (RR, 0.81; 95% CI, 0.50-1.31; p = 0.39), cardiovascular mortality (RR, 1.74; 95% CI, 0.78-3.89; p = 0.17), and myocardial infarction (RR, 0.76; 95% CI, 0.46-1.27; p = 0.30).

**Conclusions:**

PCB angioplasty demonstrated clinical and angiographic outcomes comparable to DES in SVD, supporting its role as a safe, effective alternative in selected patients.

## Introduction

Small vessel coronary artery disease (SVD) is common in patients undergoing percutaneous coronary intervention (PCI), representing up to 50% of all procedures performed annually.^1^Treating SVD with drug-eluting stents (DES) presents distinct challenges, including an increased risk of dissection, vessel rupture, and restenosis as well as the requirement for sustained dual antiplatelet therapy (DAPT) after stent implantation.^2^

Although DES have shown superiority over plain balloon angioplasty, drug-coated balloons (DCB) have emerged as a promising alternative in specific anatomical settings, including SVD.^3^DCBs are balloon catheters coated with antiproliferative agents — most commonly paclitaxel, sirolimus, or other limus analogs — that are delivered to the vessel wall during balloon inflation.^4^The balloon is typically inflated for 30-60 seconds. This strategy avoids the implantation of permanent foreign material, which may reduce late inflammatory reactions and lower the risk of restenosis and thrombosis. The lack of a metallic scaffold and durable polymer helps preserve vascular anatomy and vasomotor function, thereby minimizing hemodynamic disturbances and potentially reducing the need for sustained DAPT.^5^

Paclitaxel is cytotoxic, but it achieves cytotoxic concentrations in the vessel wall only during the first hours after DCB application. Thereafter, its activity resembles that of sirolimus. Because of its high lipophilicity, paclitaxel demonstrates greater retention in the vessel wall, resulting in more sustained inhibition of neointimal proliferation compared with limus-based drugs. This may provide a long-term advantage for paclitaxel-coated balloons (PCB) over sirolimus-coated devices.^6 , 7^

Despite expert consensus supporting DCB angioplasty as a feasible alternative to DES in SVD, evidence from randomized controlled trials (RCTs) remains limited, primarily because of small sample sizes.^8 - 11^Accordingly, we performed a systematic review and meta-analysis to synthesize available data comparing clinical outcomes of these 2 treatment strategies.

## Methods

### Eligibility criteria

Studies were eligible for inclusion if they met the following criteria: RCTs or nonrandomized cohort studies; PCI strategies involving either DCB or DES; and enrollment of patients with SVD (reference vessel diameter < 3.0 mm). Studies were included only if they reported at least 1 relevant clinical outcome.

Studies with inappropriate comparators, reference vessel diameter ≥ 3mm, follow-up shorter than 6 months, unavailable full text, or publications in languages other than English were excluded.

### Search strategy and data extraction

This systematic review and meta-analysis followed Cochrane Collaboration recommendations and was reported according to the Preferred Reporting Items for Systematic Reviews and Meta-Analyses (PRISMA) ( Table S1 ).^12 , 13^The review protocol was prospectively registered in the PROSPERO database on January 25, 2024, under the registry code CRD42024506502.

Searches were conducted by 2 review authors in PubMed, Embase, and Cochrane Library from inception to January 2024, using the terms *drug-coated balloon* , *drug-eluting stent* , and *coronary small vessel* . The complete search strategy is provided in the Supplementary Material . Reference lists were also screened for relevant studies. ClinicalTrials.gov was searched to identify additional relevant trials not captured by the primary search.

Study characteristics, baseline data, and clinical outcomes were independently extracted by 4 review authors according to predefined criteria and assessments of quality. Disagreements were resolved through discussion with 2 additional review authors and, when necessary, by consultation with the senior review authors.

### Outcomes and subanalyses

The primary outcome was the incidence of clinically driven target lesion revascularization (TLR) at the longest available follow-up. The main secondary angiographic outcome was late lumen loss (LLL). Additional outcomes of interest included major adverse cardiovascular events (MACE), all-cause mortality, cardiovascular (CV) mortality, and myocardial infarction (MI). Detailed outcome data for each study are presented in the Supplementary Material (Table S2) .

### Assessment of quality

Nonrandomized cohort studies were evaluated using the Newcastle-Ottawa Scale (NOS).^14^This tool assigns up to 9 stars based on 3 domains: selection of participants, comparability of study groups, and ascertainment of outcomes ( Table S3 ). RCTs were assessed using the Cochrane Risk of Bias Tool 2.0 (RoB 2), which classifies studies as having “low risk of bias,” “some concerns,” or “high risk of bias” across 5 domains: the randomization process, deviations from intended interventions, missing outcome data, measurement of the outcome, and selection of the reported result ( Figure S4 ).^15^

Certainty of evidence was assessed by 2 independent review authors (R.S. and M.G.) using the GRADE (Grading of Recommendations, Assessment, Development and Evaluation), with levels ranging from high to very low ( Table S5 ).^16^Publication bias was explored using funnel-plot analysis of point estimates weighted by study size and Egger’s regression test.

### Statistical analysis

Data were synthesized using a random-effects meta-analysis with a restricted maximum likelihood estimator. This model was chosen to account for potential clinical, methodological, and statistical heterogeneity, as it recognizes that the true effect of the intervention may vary across studies.^17 , 18^Binary outcomes were expressed as risk ratios (RR), and continuous outcomes as mean differences (MD). Subgroup analyses compared outcomes between randomized trials and observational studies, accounting for potential qualitative and quantitative interactions. Statistical significance was set at a 95% CI and a p-value < 0.05. Heterogeneity was assessed with chi-square, Tau, and Tau^2^tests. To avoid misleading interpretation from a fixed threshold for I^2^, heterogeneity was further evaluated in relation to the prediction interval (PI).^18 , 19^All statistical analyses were performed in R software version 4.4.1 (R Foundation for Statistical Computing).^20^

## Results

### Search strategy and baseline characteristics of the included studies

The initial search identified 566 records. After the removal of 236 duplicates, 312 articles were excluded based on title and abstract review ( Figure 1 ). A total of 18 articles underwent full-text evaluation. Of these, 2 were excluded due to unavailability of the full text, 1 for using an inappropriate comparator, and 3 for enrolling patients with a reference vessel diameter ≥ 3 mm. A total of 12 studies comprising 17,441 patients with SVD were included.^21 - 32^They were published between 2010 and 2024, with a mean follow-up of 8-36 months. All DCBs were paclitaxel-coated. Commercial names of PCBs and DES used in the individual studies are listed in the Supplementary Material (Table S6) .

**Figure 1:** – PRISMA flow diagram of study identification, screening, and inclusion. PRISMA: Preferred Reporting Items for Systematic Reviews and Meta-Analyses.

Characteristics of the included studies and interventions are summarized in Tables 1 and 2 . At baseline, the population was predominantly male, with a mean age of 64 years and a high prevalence of diabetes and hypertension. The mean reference vessel diameter was 2.19 mm. The left anterior descending artery was the most common site of target lesions. Nearly half of patients presented with multivessel disease. Left ventricular ejection fraction (LVEF) was not reported in half of the studies; in those that provided data, LVEF values were within the range consistent with preserved EF.

**Table 1:** – Baseline clinical characteristics of the included studies

Study/ Characteristic	Cortese, et al., 2010^21^	Latib, et al., 2012^22^	Sinaga, et al., 2016^27^	Giannini, et al., 2017^23^	Jeger, et al., 2018^28^	Sim, et al., 2018^24^	Tang, et al., 2018^29^	Cortese, et aI., 2020^31^	Silverio, et al., 2020^30^	Tsai, et al., 2022^26^	Kawai, et al., 2022^25^	Liu, et al., 2024^32^
Sample size, n	28/29	90/92	172/163	90/91	382/376	87/200	116/110	118/114	1154/13634	47/59	19/23	129/118
Study design	RCT	RCT	Retrospective Cohort	Retrospective Cohort	RCT	Retrospective Cohort	RCT	RCT	Prospective Cohort	Retrospective Cohort	RCT	RCT
Clinical follow-up, months	9	24	12	12	12	12	12	12	36	12	8	12
Age, years	68/67	65/66	61/61	65/66	67/68	58/61	60/60	64/66	68/69	65	69/73	60/60
Male sex, %	79/76	80/77	77/72	80/78	77/70	81/77	77/77	77/70	70/66	83/81	79/74	73/70
Diabetes, %	46/37	43/38	51/49	43/40	32/34	63/54	39/43	38/35	35/38	49/54	26/34	35/38
Hyperlipidemia, %	60/45	78/79	70/72	79/78	68/69	80/76	52/50	60/55	59/50	85/71	79/82	45/54
Hypertension, %	75/69	80/81	72/69	78/81	84/88	79/68	67/78	65/66	68/64	93/83	89/91	73/75
Current smoker, %	NA	16/10	30/44	43/33	21/19	48/29	29/32	19/16	12/16	44/35	52/52	21/21
Previous MI, %	17/20	51/35	28/27	51/58	41/35	56/57	22/25	28/39	31/24	10/11	10/21	25/22
Stable CAD, %	46/44	75/78	22/34	75/81	70/73	NA	31/30	53/56	32/23	87/81	100/100	20/25
Unstable angina, %	53/55	24/21	49/38	24/18	12/11	NA	69/73	15/15	11/10	12/18	0	63/65
NSTEMI, %	0	0	—	0	13/14	35/30	0	19/22	41/43	—	0	0
STEMI, %	0	0	28/27	0	3/1	20/27	0	10/7	14/23	NA	0	0
LVEF, %	NA	NA	NA	NA	60/60	NA	60/60	58/58	NA	58/59	67/66	62/61
Multivessel disease, %	60/65	62/60	NA	61/71	82/75	72/78	41/41	72/75	37/49	NA	0/0	0/0.8

Values are presented as means unless otherwise specified. The statistical significance level adopted in the included studies was 5%, when available in the original publications. CAD: coronary artery disease; DCB: drug-coated balloon; DES: drug-eluting stent; LVEF: left ventricular ejection fraction; MI: myocardial infarction; NA: not applicable; NSTEMI: non-ST-segment elevation MI; RCT: randomized controlled trial; STEMI: ST-segment elevation MI.

**Table 2:** – Baseline angiographic and procedural characteristics of the included studies

Study/ Characteristic	Cortese, et al., 201021	Latib, et al., 201222	Sinaga, et al., 201627	Giannini, et al., 201723	Jeger, et al., 201828	Sim, et al., 201824	Tang, et al., 201829	Cortese, et aI., 202031	Silverio, et al., 202030	Tsai, et al., 202226	Kawai, et al., 202225	Liu, et al., 2024^32^
Sample size, n	28/29	90/92	172/163	90/91	382/376	87/200	116/110	118/114	1154/13634	47/59	19/23	129/118
Angiographic follow-up, months	6	24	12	12	12	12	9	6	36	12	8	9
Left anterior descending coronary artery, %	54/52	11/11	41/41	11/17	34/31	NA	10/10	39/40	49/45	42/44	42/17	22/23
Diagonal coronary artery, %	NA	17/8	NA	17/16	NA	NA	14/15	NA	NA	NA	NA	NA
Left circumflex coronary artery, %	18/10	11/16	32/34	11/13	47/49	NA	59/47	31/37	35/32	36/43	26/61	52/48
Left main coronary artery, %	NA	NA	NA	NA	NA	NA	NA	NA	0.4/0.5	NA	NA	NA
Obtuse marginal artery, %	NA	27/32	NA	27/18	NA	NA	4/6	NA	NA	NA	NA	NA
Right coronary artery, %	28/38	8/9	26/23	8/11	20/20	NA	9/10	30/23	15/22	22/13	32/22	26/29
Posterior descending / posterolateral coronary artery, %	NA	27/22	NA	27/20	NA	NA	20/26	NA	NA	NA	NA	NA
Lesion length, mm	12.4/11.3	15.4/14.4	NA	NA	NA	18.5/20.3	10.5/10.8	13.5/14	NA	19.8/20	11.8/11.3	12.3/11.5
Diameter stenosis, %	86/89	82/83	NA	82/82	NA	88/90	70/71	75/76	NA	87/86	68/60	69/71
Minimum lumen diameter, mm	0.48/0.40	NA	NA	NA	NA	0.20/0.19	0.64/0.65	0.82/0.83	NA	0.29/0.29	0.75/0.80	0.68/0.65
Reference vessel diameter, mm	2.45/2.36	2.41/2.41	2.22/2.44	2.49/2.5	NA	1.88/1.95	2.42/2.42	2.23/2.18	2.23/2.18	2.1/2.1	2.29/2.08	2.2/2.21
Device pressure, ATM	7.7/13.4	9.6/17.2	NA	9.6/17.8	11.1/13.6	NA	9.1/10.7	13.7/11.4	10/18	NA	7.7/13	10.2/10.8
Device diameter, mm	2.48/2.54	2.49/2.49	2.25/2.5	2.49/2.5	2.75/2.57	NA	2.41/2.41	NA	2.5/2.5	2.09/2.18	2.6/2.4	2.44/2.42
Device length, mm	18.6/18.9	25.6/18.5	20.2/22.2	25.6/27.1	23.9/23.1	NA	21/20.4	21.8/18.3	20/18	26/25	19/17	20.5/19.4

Values are presented as absolute numbers (n) or percentages (%). The statistical significance level adopted in the included studies was 5% when available in the original publications. ATM: atmospheres.

### Outcomes

At a mean follow-up of 32.4 months, no statistically significant differences were found between DCB and DES for any of the analyzed outcomes. The primary outcome, TLR, occurred in 6.6% of patients in the DCB group compared with 5.3% in the DES group (RR, 1.24; 95% CI, 0.82-1.85; Figure 2A ). The secondary angiographic outcome, LLL, was also similar between groups (MD, –0.09 mm; 95% CI, –0.41 to 0.23), with mean values of 0.17 mm in the DCB group and 0.16 mm in the DES group ( Figure 2B ). Other angiographic outcomes showed no significant differences and are presented in Supplementary Figures S7A and S7B .

**Figure 2:** – Comparison of Target Lesion Revascularization and Late Lumen Loss Between Paclitaxel-Coated Balloons and Drug-Eluting Stents in Small Vessel Disease. DCB: drug-coated balloon; DES: drug-eluting stent; LLL: late lumen loss; PCB: paclitaxel-coated balloon; PI: prediction interval; RR: risk ratio; TLR: target lesion revascularization.

Clinical outcomes likewise did not differ significantly between groups. RR for MACE was 1.01 (95% CI, 0.76-1.33; Figure 3A); for all-cause mortality, 0.81 (95% CI, 0.50-1.30; Figure 3B ); for CV mortality, 1.74 (95% CI, 0.78-3.89; p = 0.17; I^2^= 0%; PI = 0.01-323.77; Figure S8 ); and for MI, 0.76 (95% CI, 0.46-1.27; Figure 3C ). Central Illustration summarizes the key findings of the review.

**Figure 3:** – Comparison of Major Adverse Cardiovascular Events, All-Cause Mortality, and Myocardial Infarction Between Paclitaxel-Coated Balloons and Drug-Eluting Stents in Small Vessel Disease. MACE: major adverse cardiovascular events; DCB: drug-coated balloon; DES: drug-eluting stent; PCB: paclitaxel-coated balloon; PI: prediction interval; RR: risk ratio.

Subgroup analyses by study design (RCTs vs. nonrandomized cohort studies) yielded consistent results.

### Assessment of quality and certainty of evidence

The risk of bias for RCTs and nonrandomized cohort studies included in this meta-analysis was considered low ( Table S4 ).

Funnel-plot analysis of the MACE outcome, used to assess potential publication bias through visual inspection of asymmetry, suggested a favorable bias toward DCB benefits ( Figure 4 ). However, Egger’s regression test did not reach statistical significance for publication bias. Complete GRADE is provided in the Supplementary Material (Table S5) .

**Figure 4:** – Funnel-plot analysis of major adverse cardiac events outcome.

## Discussion

This systematic review and meta-analysis comparing PCB and DES in SVD included 12 studies encompassing 17,441 patients, with follow-up durations ranging from 8 to 36 months (mean 32.4 months). No significant differences were observed in the primary outcome, clinically driven TLR, or in secondary outcomes such as LLL, MACE, all-cause mortality, CV mortality, and MI. These findings support the safety and efficacy of PCB as a viable alternative to DES for the treatment of SVD. The consistency of results across trials, including those using newer-generation DCBs, further strengthens this evidence.

Our pooled analysis highlighted a high prevalence of CV risk factors, with more than half of the patients presenting with hypertension and over one-third with diabetes, reflecting a real-world population undergoing PCI for SVD.

The largest trial included, BASKET-SMALL 2 (Basel Stent Kosten Effektivitäts Trial Drug Eluting Balloons vs Drug Eluting Stents in Small Vessel Interventions),^28^enrolled patients with successful lesion pre-dilatation and reported no significant difference in MACE at 12 months between DCB and DES groups (7.3% vs 7.5%; hazard ratio, 0.97; 95% CI, 0.58-1.64; p = 0.92) as well as similar rates of cardiac death, MI, and target vessel revascularization. By contrast, the PICCOLETO II (Drug Eluting Balloon Efficacy for Small Coronary Vessel Disease Treatment) trial^31^demonstrated superiority of DCB over DES in terms of in-lesion LLL at 6 months. Long-term follow-up showed similar mortality and MI rates, but higher MACE and acute vessel occlusion in the DES group (20.8% vs 10.8%, *p* = 0.046; and 4% vs 0%, *p* = 0.042, respectively).^33^The REC-CAGEFREE I (Drug-Coated Balloon Angioplasty With Rescue Stenting Versus Intended Stenting for the Treatment of Patients With de Novo Coronary Artery Lesions) trial^34^did not establish overall noninferiority of PCB; however, outcomes in the SVD subgroup (device diameter < 3.0 mm) were comparable ( *p* -value for interaction = 0.02).

Although current guidelines recommend DES over DCB for in-stent restenosis,^35^the use of DCB for de novo lesions in small vessels has gained traction in clinical practice, particularly among patients at high bleeding risk. While not yet incorporated into major guidelines, this approach is endorsed by several expert consensus statements.^8 - 10^A key advantage of DCB is the possibility of shorter DAPT, as brief as 30 days, thereby reducing bleeding risk^36^without increasing MACE.^37^In addition, the lack of a permanent implant may decrease the incidence of acute and subacute thrombosis. Nevertheless, further evidence is required to confirm long-term safety and efficacy.

Findings regarding bleeding risk remain inconsistent. Jeger et al.^28^reported a 2-fold increase in bleeding with DES at 1 year, whereas Cortese et al.^21^found no significant differences between devices. In the DEBUT trial, which investigated high bleeding risk patients treated with only 1 month of DAPT, DCB was associated with lower CV death and MI rates, although bleeding rates were similar at 9 months.^38^Importantly, bleeding outcomes were inconsistently reported across the studies included in this meta-analysis, precluding a pooled analysis.

Restenosis continues to be a concern with DCB. A propensity-adjusted analysis from the SCAAR (Swedish Coronary Angiography and Angioplasty Registry) trial found higher restenosis rates with DCB; however, no differences were seen in hard outcomes such as target lesion thrombosis, MI, or death.^30^Similarly, Latib et al.^22^reported comparable outcomes in a RCT with 2-year follow-up. Because of inconsistent reporting, restenosis was not analyzed in this meta-analysis.

DCBs may also be used as part of a hybrid strategy, particularly in long or diffuse lesions. In this approach, DES are typically implanted in large vessels with residual disease after balloon angioplasty, whereas DCBs are reserved for small vessels, which are more prone to restenosis, impaired vasomotion, neoatherosclerosis, and future challenges in coronary artery bypass grafting.^1 , 39^

Although all PCBs deliver paclitaxel, they differ in formulation and device characteristics, such as the excipient (eg, iopromide [SeQuent^®^Please, B. Braun] vs. urea [IN.PACT™ Falcon™, Medtronic]) and balloon profile. Hydrophilic excipients like urea may facilitate drug transfer and could be advantageous in short, simple lesions.^40^Device flexibility and deliverability also vary, potentially influencing procedural success in complex anatomy.

This meta-analysis has several limitations. Most included studies did not perform angiographic follow-up, restricting the analysis of LLL and restenosis. As a result, the study by Silverio et al.^30^, the largest in this meta-analysis, was excluded from the TLR outcome due to unavailable data. In addition, 11 of the 12 studies used second-generation DES, a factor that should be considered when extrapolating these results.^21^Another limitation is the lack of a universally accepted definition for SVD, as diameter thresholds varied across studies. Finally, the limited number of RCTs required inclusion of nonrandomized cohort studies. While RCTs provide greater internal validity, observational data contribute valuable information on long-term outcomes.

## Conclusion

This systematic review and meta-analysis, encompassing 12 studies, found no statistically significant difference between PCBs and DESs in the treatment of SVD. These findings support the potential role of DCBs as a safe, effective alternative to stents in the treatment of SVD. However, further RCTs with longer follow-ups are warranted to confirm these results and guide their broader adoption in routine clinical practice, particularly for de novo lesions in small coronary vessels.

## Supplemental Materials

SUPPLEMENTAL MATERIAL
